# Expanding the clinical spectrum of *COL2A1* related disorders by a mass like phenotype

**DOI:** 10.1038/s41598-022-08476-7

**Published:** 2022-03-16

**Authors:** Till Joscha Demal, Tasja Scholz, Helke Schüler, Jakob Olfe, Anja Fröhlich, Fabian Speth, Yskert von Kodolitsch, Thomas S. Mir, Hermann Reichenspurner, Christian Kubisch, Maja Hempel, Georg Rosenberger

**Affiliations:** 1 Department of Cardiovascular Surgery, University Heart & Vascular Center Hamburg, Hamburg, Germany; 2 Institute of Human Genetics, University Medical Center Hamburg-Eppendorf, Hamburg, Germany; 3 Department of Cardiology, University Heart & Vascular Center Hamburg, Hamburg, Germany; 4 Pediatric Cardiology Clinic, University Heart & Vascular Center Hamburg, Hamburg, Germany; 5 Department of Paediatrics, University Medical Center Hamburg-Eppendorf, Hamburg, Germany; 6 Present Address: Genetic Clinic, Institute of Human Genetics, Heidelberg University Hospital, Heidelberg, Germany

**Keywords:** Medical genetics, Cardiovascular biology, Interventional cardiology, Genetics, Disease genetics

## Abstract

MASS phenotype is a connective tissue disorder clinically overlapping with Marfan syndrome and caused by pathogenic variants in *FBN1*. We report four patients from three families presenting with a MASS-like phenotype consisting of tall stature, arachnodactyly, spinal deformations, dural ectasia, pectus and/or feet deformations, osteoarthritis, and/or high arched palate. Gene panel sequencing was negative for *FBN1* variants. However, it revealed likely pathogenic missense variants in three individuals [c.3936G > T p.(Lys1312Asn), c.193G > A p.(Asp65Asn)] and a missense variant of unknown significance in the fourth patient [c.4013G > A p.(Ser1338Asn)] in propeptide coding regions of *COL2A1*. Pathogenic *COL2A1* variants are associated with type II collagenopathies comprising a remarkable clinical variablility. Main features include skeletal dysplasia, ocular anomalies, and auditory defects. A MASS-like phenotype has not been associated with *COL2A1* variants before. Thus, the identification of likely pathogenic *COL2A1* variants in our patients expands the phenotypic spectrum of type II collagenopathies and suggests that a MASS-like phenotype can be assigned to various hereditary disorders of connective tissue. We compare the phenotypes of our patients with related disorders of connective tissue and discuss possible pathomechanisms and genotype–phenotype correlations for the identified *COL2A1* variants. Our data recommend *COL2A1* sequencing in *FBN1*-negative patients suggestive for MASS/Marfan-like phenotype (without aortopathy).

## Introduction

**Figure 1:**
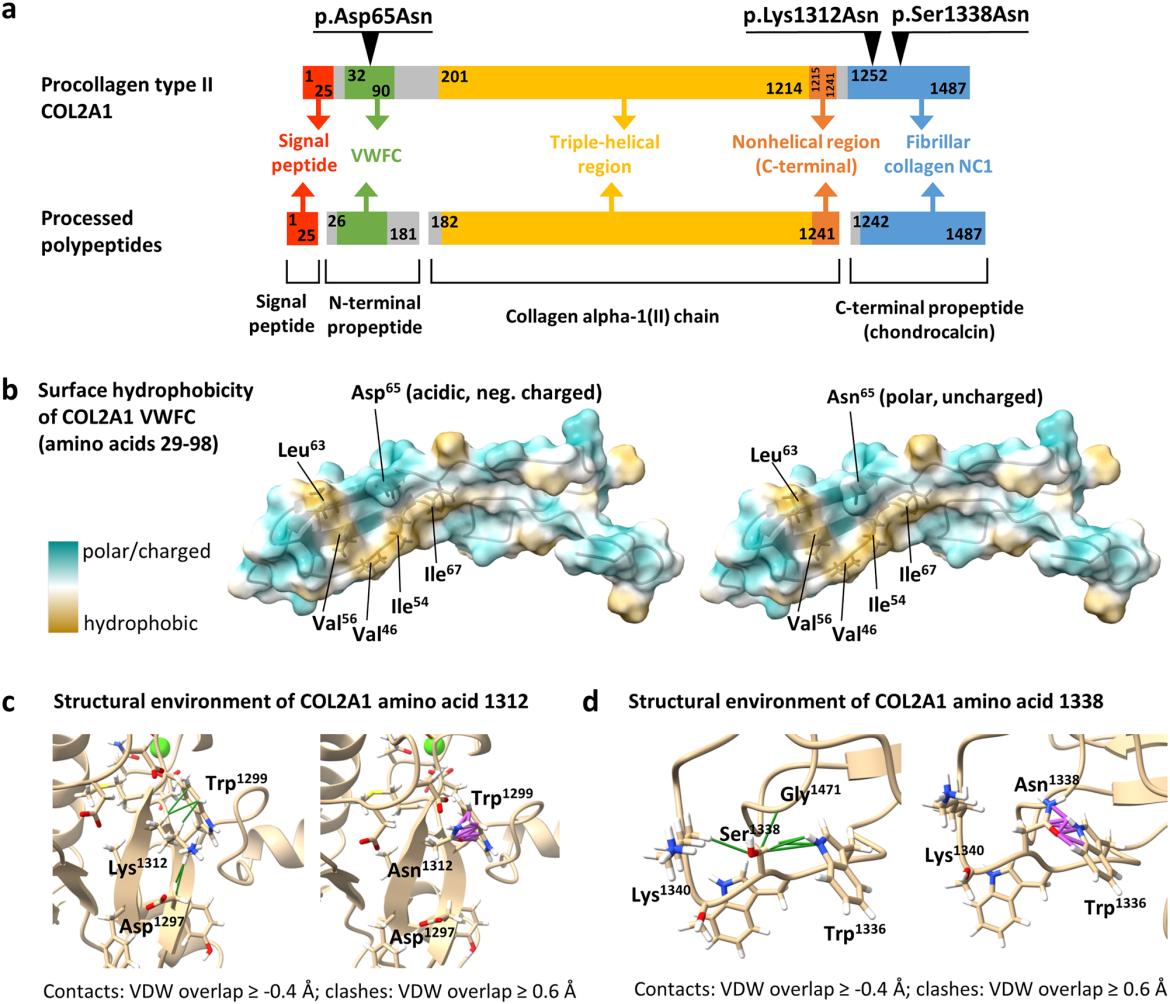
(**a**) Schematic representation of COL2A1 protein domains, regions and peptides as well as molecular processing products. The size of protein domains and regions is indicated by amino acid positions within the schema. The localisation of disease-associated COL2A1 amino acid changes p.(Asp65Asn) in the VWFC repeat as well as p.(Lys1312Asn) and p.(Ser1338Asn) in the fibrillar collagen NC1 domain is shown. VWFC, von Willebrand factor (VWF) type C repeat; fibrillar collagen NC1, fibrillar collagen C-terminal non-collagenous (NC1) domain. (**b**) Visualisation of surface hydrophobicity of the COL2A1 VWFC domain (amino acids 29–98). COL2A1 Asp^65^ affected in subject 2 and amino acids Val^46^, Ile^54^, Val^56^, Leu^63^, and Ile^67^ are labeled (left model). The right model shows the surface hydrophobicity after molecular replacement of Asp^65^ by asparagine. Different colours indicate the hydrophobicity properties of amino acids. The most polar and charged residues are in cyan and the most hydrophobic residues are in tan. The surface colouring feature of the UCSF Chimera tool (version 1.2) was used^61^. VWFC, von Willebrand factor (VWF) type C repeat. (**c**) Structural impact of the COL2A1 p.Lys1312Asn amino acid change. Ribbon representations of the COL2A1 fibrillar collagen NC1 domain show amino acids 1312 and surrounding residues within a radius of 5 Å as well as calcium (green sphere) binding residues as sticks. Sidechains are coloured by element (hydrogen: white; carbon: beige; oxygen: red; nitrogen: blue; cysteine: yellow). The left model shows the structural environment of Lys^1312^ with Van-der-Waals (VDW) overlaps ≥ -0.4 Å (contacts, green lines), whereas the right model depicts the structural environment of Asn^1312^ with VDW overlaps ≥ 0.6 Å (clashes, magenta lines). (**d**) Structural impact of the COL2A1 p.Ser1338Asn amino acid change. Ribbon representations show details of the COL2A1 fibrillar collagen NC1 domain for wild-type (p.Ser1338, left) and mutated (p.Asn1338, right) COL2A1. Colour codes and prediction of VDW contacts and clashes are as mentioned above.

The *COL2A1* gene encodes procollagen type II. Three of these molecules form the triple-helical procollagen homotrimer^1^. After secretion into the extracellular matrix (ECM) and removal of procollagen's N- and C-terminal propeptides (Fig. 1a), processed collagens form a covalently cross-linked fibrillar network in the ECM, that provides tensile strength to connective tissues^2^. Type II collagen is found primarily in cartilage, the adult vitreous, the pituitary gland, the stomach, and the epididymis (proteinatlas.org and gtexportal.org, accessed 06-29-2021); it is synthesized mainly by chondrocytes. Pathogenic sequence variants in the *COL2A1* gene cause clinically distinguishable type II collagenopathies with mild to lethal phenotypes, which are usually of dominant inheritance^3,4^. Main known features of *COL2A1*-associated disorders are skeletal dysplasia, ocular anomalies, and auditory impairment, all with different severities^3^. The skeletal manifestations are extremely heterogeneous itself and range from osteoarthritis (OA) with mild chondrodysplasia (OSCDP, *MIM* #604,864) to achondrogenesis type II (ACG2, *MIM* #200,610) and platyspondylic skeletal dysplasia, Torrance type (PLSD; *MIM* #151,210) with potentially fatal courses^4,5^. Ocular symptoms are diverse and include myopia, retinal detachment, lattice degeneration of the retina, cataracts and glaucoma^6^. Auditory manifestations mainly include sensorineural hearing loss; conductive hearing loss may occur, especially in children and patients with palatal defects^7^.

Various types of *COL2A1* gene alterations (e.g. substitutions, deletions) primarily with missense and loss-of-function consequences have been described^8–11^. Most disease-associated *COL2A1* variants (i.e. 80–85%) are located in the triple-helical region of the collagen chain (Fig. 1a)^4,11^. In this region, missense variants have been associated with severe phenotypes [e.g. ACG2, Kniest dysplasia (*MIM* #156,550), Spondyloepiphyseal dysplasia (congenital type, SEDC, *MIM* #183,900)], whereas loss-of-function variants (e.g. nonsense variants, splice site variants, frameshift variants) have been mainly described in patients with milder phenotypes such as Stickler syndrome, type 1 (STL1)^4,8,9^. Thus, the molecular spectrum is different across the phenotypes^8^. The relationship between phenotypes and variants in the terminal regions of *COL2A1* as well as propeptide-specific pathomechanisms are poorly understood; even if variants in the N- and C-propeptides (Fig. 1a) have been associated with milder and severe/lethal phenotypes, respectively^4^.

Here, we report four patients carrying variants in the propeptide regions of *COL2A1* and presenting with tall stature, arachnodactyly, dural ectasia, and varying skeletal manifestations, resembling MASS (mitral valve, myopia, aorta, skeletal and skin features) phenotype.

## Material and methods

### Proband recruitment and clinical examination

Probands presented at the University Heart and Vascular Center Hamburg in our specialized outpatient clinic for connective tissue diseases or in the pediatric outpatient clinic for hereditary aortopathies between 12/2017 and 09/2021. Subsequent standardized clinical examination, 200 patients were included to this study according to following criteria: clinical features were suggestive of Marfan syndrome (MFS), Loeys-Dietz syndrome (LDS), Ehlers-Danlos syndrome (EDS), congenital contractural arachnodactyly (CCA), MASS phenotype, syndromic/non-syndromic TAAD, or an unspecified heritable disorder of connective tissue with or without vascular involvement. Blood samples of these 200 probands were obtained and targeted next-generation sequencing (tNGS, i.e. gene panel sequencing) was performed. The four subjects described in this study were referred to the clinical genetics outpatient clinic of the University Medical Center Hamburg-Eppendorf for genetic counselling and further clinical examination. They underwent clinical scoring on Marfan syndrome according to the revised Ghent nosology^12^. Aortic root measurements were carried out according to the Ghent nosology guidelines (for details see Supplementary Information). Spinal MRI scans were performed in 3 individuals to screen for dural ectasia.

### Genetic testing, variant prioritization and classification

In each patient, 62 genes were analyzed using a tNGS approach as previously reported by our group^13^. These genes are either associated with thoracic aortic aneurysm/dissection-spectrum disorders or with connective tissue diseases or they have crucial functions in connective tissue homeostasis (Table S1). Synonymous, missense and nonsense variants, coding indels, and intronic alterations at exon–intron boundaries ranging from −10 to + 10 were included into the analysis. Variants with an allele frequency (AF) according to the gnomAD database v.2.1.1^14^ higher than the predicted maximum population frequency (MPF) of respective gene variants were excluded from further analysis. The MPF was calculated based on the respective disease prevalence, penetrance, and the genetic/allelic contribution of the respective gene/disease^15^, which resulted in an MPF of 5.15E-06 for *COL2A1* variants (Table S1). Variants passing these filters were classified according to their likelihood for pathogenicity based on the American College of Medical Genetics and Genomics and the Association for Molecular Pathology (ACMG/AMP) guidelines on variant interpretation: pathogenic variant (PV), likely pathogenic variant (LPV), variant of uncertain significance (VUS), likely benign variant (LBV), and benign variant (BV)^16,17^. Individual ACMG/AMP criteria were assigned according to an automated *in-silico* VarSome analysis (version 10.2)^18^. Criteria based on *in-silico* splicing prediction and familial segregation were added manually. Reportable variants including (L)PV and VUS were communicated to the subjects. Detailed information on targeted next-generation sequencing, variant prioritization and classification, concomitant variants as well as molecular modelling is given in the Supplementary Information.

## Results

**Figure 2:**
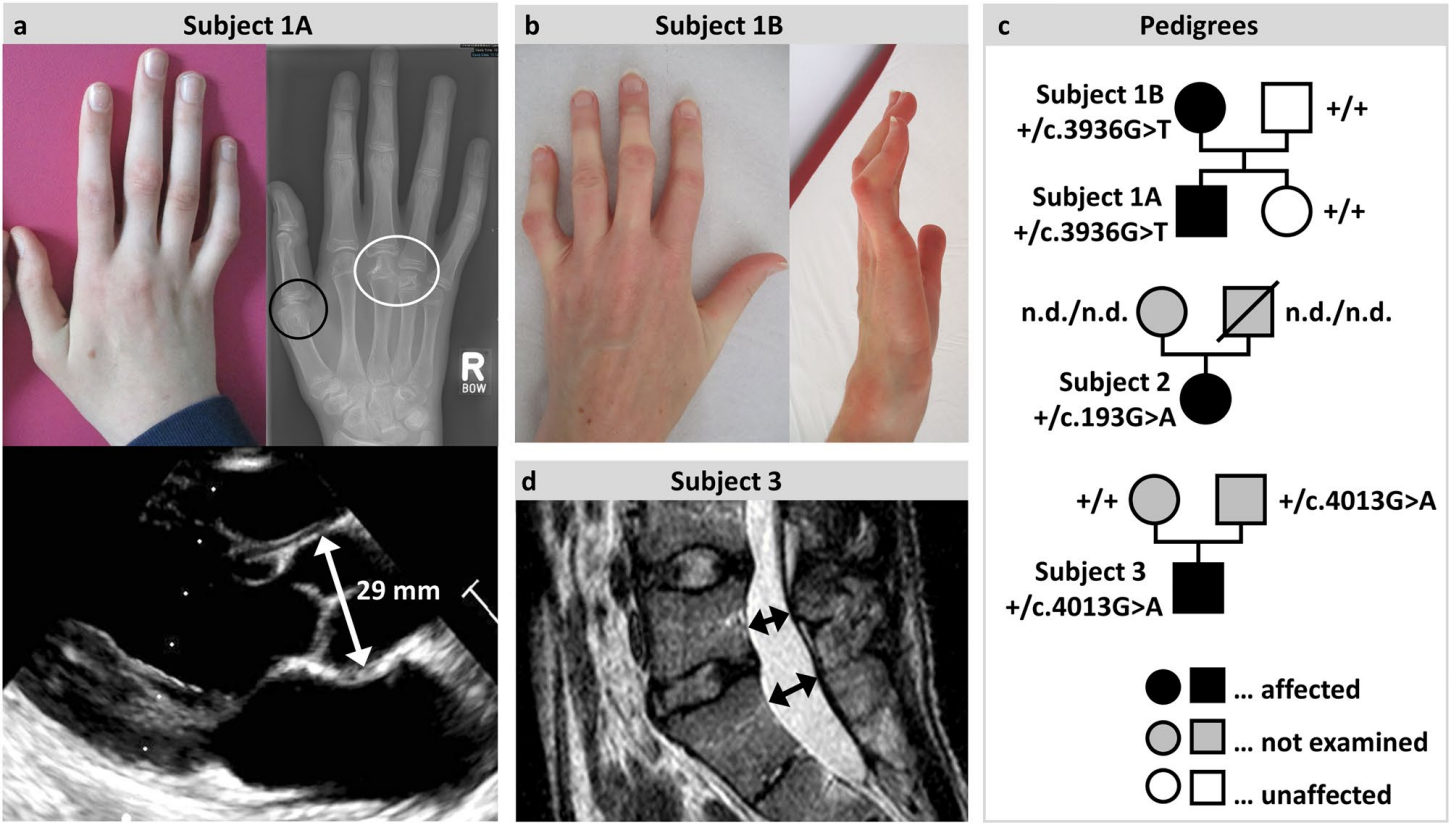
Selected clinical features of the presented patients. (**a**) Photographs, X-ray image and transthoracic echocardiography in parasternal long-axis of subject 1A. Note the long and slender fingers (arachnodactyly) and camptodactyly of digit V of the right hands (upper left image) as well as the subluxation of the proximal phalanx of the right thumb (black circle, upper right image) and erosive changes of the distal epiphysis of the 3^rd^ and 4^th^ metacarpal bone (white circle, upper right image). The lower image shows normal aortic root diameter of 29 mm (Z-Score 1.3) and intact aortic shape. (**b**) Photographs of subject 1B. Note the long and slender fingers (arachnodactyly) with camptodactyly of the fifth finger. (**c**) Pedigrees of the families of subjects 1, 2 and 3. Affected individuals show a MASS-like phenotype and carry a putative disease-relevant *COL2A1* variant. Identified heterozygous variants in *COL2A1* are given. Parents of subjects 2 and 3 were not clinically examined and segregation analysis was not possible in the parents of subject 2. + , wild-type allele; n.d., not determined. **(d)** Magnetic resonance images of the lower spine and spinal cord of subject 3. Note the lumbosacral dural ectasia (black arrows).

Subject 1A was first examined in our clinic at the age of 11 years. He was born after uncomplicated pregnancy and underwent pyloromyotomy due to hypertrophic pyloric stenosis in the newborn period. Otherwise, he was a healthy boy with a normal development. The family history was positive for arthritis/gout but negative for aortic aneurysms/dissections and sudden cardiac deaths. At the age of 9 years, he developed painless swelling of the metacarpophalangeal joints (MCP) of the right hand. Juvenile idiopathic arthritis (JIA) was suspected by the age of 10 years. An MRI scan of the hand revealed synovitis and effusion of the MCP3 joint. Antinuclear antibodies (ANA) were positive (maximum titer 1:2,560), whereas rheumatoid factors and Human Leukocyte Antigen (HLA) B27 could not be detected. The patient showed progressive arthritis of further MCP joints of both hands and the wrist of the right hand. He was treated with nonsteroidal anti-inflammatory drugs (NSAID) and methotrexate. Further MRI scans showed necrosis of the medial sesamoid bone of the left first toe and bursitis trochanterica as well as mild synovialitis of the left hip joint. At the age of 11 years, the clinical examination revealed arachnodactyly (Fig. 2a) with positive wrist and thumb signs. X-ray of his hands detected arthritis-associated destruction of the metacarpophalangeal joints of the fingers and the distal radioulnar joint. Subluxation of the carpometacarpal joints of both thumbs was observed (Fig. 2a). In consequence, the therapy was extended by Adalimumab (TNF-α monoclonal antibody). One year later, no clinical improvement was observed and the treatment was changed to Etanercept (TNF-α inhibitor). Furthermore, at the age of 13 years the clinical examination revealed kyphoscoliosis and pectus excavatum. His height exceeded the 99th percentile in the toddler period and reached 178 cm (+ 1.8 SD, > 95P) by the age of 13 years^19^. His arm span to height ratio was 1.07. An MRI scan of the spine revealed dural ectasia, anterospondylolisthesis, and vertebral osteochondrosis. Significant aortic root dilatation was excluded by transthoracic echocardiography (Fig. 2a). Repeated ophthalmologic investigations remained unremarkable. The diagnosis of hereditary connective tissue disorder (HCTD) was considered and discussed. According to the revised Ghent nosology^12^, this patient reaches a systemic score of 7, which led to the diagnosis of a MASS-like phenotype (Table 1). Gene panel sequencing revealed the heterozygous missense variant c.3936G > T p.(Lys1312Asn) in *COL2A1* (Table 1), which was classified as LPV (class 4) according to the ACMG/AMP guidelines^17^. Rules, considerations and data for assigning classification criteria are outlined in the Supplementary Information (Supplementary Results and Table S2).

**Table 1:** Patients, variant classification and clinical features.

Patient	Subject 1A	Subject 1B	Subject 2	Subject 3
Sex	Male	Female	Female	Male
Age at last examination	13 years	44 years	53 years	22 years
Family history	Positive: grandfather with arthritis/gout	Positive: father with arthritis/gout	Uncertain: father with suspected arthritis	Uncertain: parents n.a. for clinical examination
**Variant classification**				
*COL2A1* (NM_001844.5) variant (c. notation, p. notation)	c.3936G > T (het) p.(Lys1312Asn)	c.3936G > T (het) p.(Lys1312Asn)	c.193G > A (het) p.(Asp65Asn)	c.4013G > A (het) p.(Ser1338Asn)
gnomAD v.2.1.1 total population allele frequency (allele count/allele number/hom)	0.000003976 (1/251,478/0)	0.000003976 (1/251,478/0)	0 (0/280,928/0)	0 (0/251,496/0)
VarSome pathogenicity prediction (damaging/uncertain/tolerated)	13/1/2	13/1/2	11/1/4	3/2/11
HGMD/ClinVar	N.l./N.l.	N.l./N.l.	N.l./VUS*	N.l./N.l.
ACMG/AMP classification (Criteria)	LPV *(PM1, PM2*^†^*, PP2, PP3)*	LPV *(PM1, PM2*^†^*, PP2, PP3)*	LPV *(PM1, PM2, PP2, PP3)*	VUS *(PM1, PM2, PP2, BP4)*
**Clinical features**				
Height	178 cm (+ 1.8 SD, > 95P)	172 cm (+ 1.3 SD, > 90P)	179 cm (+ 2.4 SD, > 95P)	198 cm (+ 3.0 SD, > 95P)
Tall stature	Yes	Yes	Yes	Yes
Arm span/arm span to height ratio	191 cm/1.07	185 cm/1.07	N.a.	N.a.
Arachnodactyly	Yes	Yes	Yes	Yes
Wrist and thumb sign	**Both positive (SySc. 3)**	**Positve wrist sign (SySc. 1)**	**Both positive (SySc. 3)**	**Positive thumb sign (SySc. 1)**
Dural ectasia	**Yes (SySc. 2)**	N.a.	**Yes (SySc. 2)**	**Yes (SySc. 2)**
Pectus deformity	**P. excavatum (SySc. 1)**	**P. excavatum (SySc. 1)**	No	**P. carinatum (SySc. 2),** Asymmetric thorax
Spinal deformity	**Kyphosis (SySc. 1)**	**Kyphosis (SySc. 1)**	**Scoliosis (SySc. 1)**	**Scoliosis (SySc. 1)**
Foot deformity	No	**Pes planus (SySc. 1)**	**Pes planus (SySc. 1)**	**Hindfoot deformity (SySc. 2)**
Aortopathy	No (ARD 29 mm, Z-score 1.3)	No (ARD 32 mm, Z-Score 0.4)	No (ARD 33 mm, Z-score 0.68)	No (ARD 36 mm, Z-Score 1.2)
Craniofacial features	High arched palate	**Dolichocephaly, enophthalmus malar hypoplasia**, high arched palate **(SySc. 1)**	No	**Dolichocephaly, malar hypoplasia, retrognathia**, high arched palate **(SySc. 1)**
Osteoarthritis	Yes	Yes	No	No
Other features	anterospondylolisthesis, vertebral osteochondrosis	bone odema of both feet	hypermobility of knee and elbow joints, varicosis	suspected bicuspid aortic valve, myopia (< 3dpt)
Diagnosis according to revised Ghent nosology	MASS-like (Systemic score 7)	MASS-like (Systemic score 5)	MASS-like (Systemic score 7)	MASS-like (Systemic score 9)

Patients, variant classification and clinical features. Variant details and clinical features are listed by patient. Nucleotide numbering uses + 1 as the A of the ATG translation initiation codon in the reference sequence, with the initiation codon as codon 1. gnomAD, Genome Aggregation Database (v2.1.1); allele frequency in total population is given; allele count/allele number/hom, total number of alleles/total number of analysed alleles/number of homozygous carriers. HGMD, Human Gene Mutation Database. ClinVar, database on the relationships between human variations and phenotypes. Variants were classified as recommended by ACMG/AMP: VUS, variant of uncertain significance or with conflicting interpretations of pathogenicity; LPV, likely pathogenic variant. Clinical features supporting a MASS-like phenotype are shown in bold and the respective systemic score (SySc.) according to the revised Ghent nosology is given in parentheses^12^. het, heterozygous; N.a., not available; N.l., not listed; SD, standard deviation; ARD, aortic root diameter; MASS, Myopia, mitral valve prolapse, borderline and non-progressive aortic root dilatation, skeletal findings and striae.

*, variant c.193G > A p.(Asp65Asn) has previously been reported in the ClinVar database and classified as VUS. The patient’s condition and the inheritance were not recorded.

^†^, VarSome 10.2 assigns PM2 for dominant genes if the allele count of the variant is less than 5 in the gnomAD database (for details see Supplementary Results).

Subject 1B is the mother of subject 1A, who presented for genetic counselling at the age of 44 years. She had a medical history of swollen finger joints since the toddler age and developed contractures of the MCP and proximal interphalangeal (PIP) joints as a teenager. She was suspected of having rheumatoid arthritis and tendinitis. However, she never experienced pain in her joints nor was treated by permanent medication. In adulthood, an MRI of the feet showed bone edema at the left Os metatarsale 3 and 4 and the right Os cuniforme, as well as mild arthrosis of the metatarsophalangeal joint of the right big toe. Echocardiography as well as ophthalmologic investigations yielded normal results. At the age of 44 years her height was 172 cm (+ 1.3 SD) and her arm span to height ratio was 1.07. Examination revealed high arched palate, pectus excavatum, scoliosis, arachnodactyly and camptodacyly of fingers II, III, and V of both hands (Fig. 2b). Facial features included dolichocephaly, enopthalmus, and malar hypoplasia. According to the revised Ghent nosology^12^, she reaches a systemic score of 5, and thus, a MASS-like phenotype was diagnosed. Genotyping showed that she carries the familial heterozygous c.3936G > T p.(Lys1312Asn) variant in *COL2A1* (Table 1). Genotyping did not identify the c.3936G > T p.(Lys1312Asn) variant in the unaffected sister and father of subject 1A (Fig. 2c).

Subject 2 underwent clinical examination in our center at the age of 53 years. The family history was negative for aortic aneurysms/dissections and sudden cardiac deaths, however, arthritis was suspected in the father. She presented with tall stature (179 cm, + 2.4 SD), arachnodactyly, scoliosis, hypermobility of knees and elbows, varicosis, and pes planus. Furthermore, she reported ear murmur of unknown cause. A lumbar spine MRI revealed dural ectasia. Echocardiographic investigation was normal with an aortic root Z-score < 2. The diagnosis of HCTD was considered and discussed. According to the revised Ghent nosology^12^, she reached a systemic score of 7, and a MASS-like phenotype was diagnosed. Gene panel sequencing revealed the heterozygous missense variant c.193G > A p.(Asp65Asn) in *COL2A1* (Table 1), which was classified as LPV according to the ACMG/AMP guidelines^17^. Rules, considerations and data for assigning classification criteria are outlined in the Supplementary Information (Supplementary Results and Table S2). Segregation analysis in parents was not possible because the father had been deceased and the mother was not available (Fig. 2c).

Subject 3 underwent clinical examination in our center at the age of 22 years. The family history was negative for connective tissue disorders, aortic aneurysms/dissections, and sudden cardiac deaths. He presented with tall stature (198 cm, + 3.0 SD), dolichocephaly, malar hypoplasia, retrognathia, high-arched palate, scoliosis, pectus carinatum with asymmetric thorax, arachnodactyly and hindfoot deformity. Ophthalmological investigation revealed iris transillumination and myopia (< 3 dpt). Bicuspid aortic valve was suspected by transthoracic echocardiography. A lumbar MRI scan showed dural ectasia (Fig. 2d). The diagnosis of HCTD was considered and discussed. The diagnosis of a MASS-like phenotype was established in this patient as well, as he has an aortic root Z-score < 2 and a systemic score of 9 according to the revised Ghent nosology^12^. Gene panel sequencing revealed the heterozygous missense variant c.4013G > A p.(Ser1338Asn) in *COL2A1* (Table 1), which was classified as VUS (class 3) according to the ACMG/AMP guidelines^17^. Rules, considerations and data for assigning classification criteria are outlined in the Supplementary Information (Supplementary Results and Table S2). The parents of subject 3 provided us with self-collected buccal swabs, but they were not available for clinical examination. Genotyping of parental DNA identified the heterozygous *COL2A1* c.4013G > A transition in the father but not in the mother of subject 3 (Fig. 2c).

Taken together, we ascertained four individuals from three families with a MASS-like phenotype and putative disease causing *COL2A1* variants. The mean age of these four subjects was 33 ± 18.6 years with an even female to male ratio. All patients presented with tall stature, arachnodactyly and (kypho)scoliosis. Dura ectasia could be detected in all three patients who underwent spine MRI. Other than that, patients presented with further marfanoid features including pectus and foot deformities and typical craniofacial dysmorphism. None of the patients showed aortic root dilatation or a specific eye phenotype (i.e. ectopia lentis).

A MASS or Marfan-like phenotype (here defined as arachnodactyly and scoliosis without aortic dilatation and ectopia lentis) was recognized in 23 (11.5%) of a total of 200 probands who underwent gene panel sequencing. The three probands (subjects 1A, 2 and 3) positive for LPV/VUS in *COL2A1* account for 13.0% (3/23) of patients with MASS/Marfan-like phenotypes (subject 1B was genotyped separately as part of a family segregation analysis). The remaining 20 probands with MASS/Marfan-like phenotypes did not show any *COL2A1* variant. However, two patients had (likely) pathogenic variants in *FBN1* and one patient had a likely pathogenic variant in *FBN2*. In another five patients of this subset, we identified eight VUS in the genes *TNXB* (3), *B4GALT7* (1), *COL5A1* (1), *BGN* (1), *MYLK* (1), and *FLNA* (1). These data suggest genetic heterogeneity of MASS/Marfan-like phenotypes.

All identified COL2A1 variants are located in the N- or the C-propeptide regions (Fig. 1a), which are removed during the processing from procollagen to collagen. The p.(Asp65Asn) substitution affects the von Willebrand factor type C (VWFC) repeat in the N-propeptide region of COL2A1 and the p.(Lys1312Asn) and p.(Ser1338Asn) substitutions are located in the fibrillar collagen C-terminal non-collagenous (NC1) (fibrillar collagen NC1) domain within the C-terminal propeptide (Fig. 1a).

We explored the structural impact of the p.Asp65Asn change by using the crystallographic structure of the COL2A1 VWFC domain (amino acids 29–98) as a template (PDB ID 5NIR)^20^. Molecular replacement of Asp^65^ for an asparagine did not significantly affect intramolecular interactions and no Van-der-Waals (VDW) overlaps (non-covalent clashes) of Asn^65^ with adjacent residues were predicted (Fig. S1a). Modelling of hydrophobicity and electrostatic potential of COL2A1 VWFC surface uncovered a hydrophobic patch comprising Val^46^, Ile^54^, Val^56^, Leu^63^ and Ile^67^ opposed by the negatively charged Asp^65^ on one side (Fig. 1b, Fig. S1b). This hydrophobic pocket mediates binding with bone morphogenetic protein 2 (BMP2)^20^. The substitution of the negatively charged Asp^65^ by a polar, uncharged asparagine may affect the binding properties and electrostatic potential of the hydrophobic interface (Fig. 1b, Fig. S1b).

For modelling the structural environment of Lys^1312^ and Ser^1338^, the crystallographic structure of COL1A1 C-terminal propeptide homo-trimer (amino acids 1218–1464) was used as template (PDB ID 5K31)^21^. SWISS-Model was applied to build a COL2A1 model based on 68.72% sequence identity with COL1A1^22^. Molecular replacement of amino acid Lys^1312^ for an asparagine resulted in loss of five intramolecular interactions (VDW contacts) with Asp^1297^ and Trp^1299^ and the formation of several potential non-covalent (i.e. VDW) clashes between the side chains of Asn^1312^ and Trp^1299^ (Figs. 1c, S2a). New VDW contacts of Asn^1312^ with adjacent residues were not predicted (Figs. 1c, S2a). However, p.Lys1312Asn replacement changed surface hydrophobicity and electrostatic potential (Fig. S2b). Molecular replacement of Ser^1338^ for an asparagine resulted in loss of six intramolecular interactions with Lys^1340^, Trp^1336^ and Gly^1471^ and the formation of several possible VDW clashes of Asn^1338^ with Trp^1336^ (Figs. 1d, S3a). New VDW contacts of Asn^1338^ with adjacent residues were not predicted (Fig. S3a) and surface hydrophobicity and electrostatic potential were unchanged (Fig. S3b). Taken together, these data suggest that both p.(Lys1312Asn) and p.(Ser1338Asn) in *COL2A1* induce structural alterations which possibly interfere with COL2A1 function.

## Discussion

### Clinical aspects

Here we report four subjects with a clinical presentation partially overlapping with Marfan syndrome (MFS; *MIM* #154700) and to a minor extent with Loeys-Dietz syndrome 3 (LDS3; syn. aneurysms-osteoarthritis syndrome; *MIM* #613795)^23,24^ (Table 2) and fulfilling the criteria of MASS-like phenotype diagnosis according to the revised Gent nosology. However, we detected no VUS, LPV or PV in the respective disease genes *FBN1* and *SMAD3*. Instead, we identified a *COL2A1* variant putatively relevant to the disease in each of the patients. It has been demonstrated that heterozygous pathogenic *FBN1* variants underlie the MASS phenotype in a total of 36 unrelated individuals^25–29^. On the other hand, *FBN1* sequencing did not reveal pathogenic variants in 17 unrelated patients with MASS phenotype^30–33^. Accordingly, it was suggested that additional disease genes for the MASS phenotype remain to be identified^34^. We detected disease-relevant *FBN1* variants only in two (8.7%) out of 23 patients with MASS/Marfan-like phenotypes. Additionally, we identified putative causal *COL2A1* variants in 3 (13.0%) individuals. To our knowledge, this is the first time that a MASS-like phenotype has been associated with causal variants in any gene other than *FBN1*.

**Table 2:** Comparison of the clinical presentation in our patients with related connective tissue disorders.

Disorder	TYPE II collagenopathies				Related syndromes	
	MASS-like phenotype	STL1	OSCPD	Czech Dysplasia	MFS	LDS3 (AOS)
References	this report	^36^	^62,63^	^64–70^	^23^	^24^
Number of patients	n = 4	n = 107	n = 54	n = 45	n = 1,013	n = 27
Disease gene	*COL2A1*	*COL2A1*	*COL2A1*	*COL2A1*	*FBN1*	*SMAD3*
**Main features in the presented cohort (%)**						
Tall stature	100	0	0	rep., r.u.	rep., r.u.	not rep.
Arachnodactyly	100	rep., r.u.	not rep.	not rep.	78	53
Spine deformity	100	rep., r.u.	rep., r.u.	rep., r.u.	53	43
Dural ectasia	100 (3/3)	not rep.	not rep.	not rep.	53	50
Dolichostenomelia	100 (2/2)	not rep.	not rep.	2	55	26
Abnormal palate	75	31	0	2	69	42
Pectus deformity	75	rep., r.u.	not rep.	not rep.	59	16
Foot deformity	75	not rep.	not rep.	rep., r.u.	47	100
Osteoarthritis	50	rep., r.u.	100	100	rep., r.u.	100
Dolichocephaly	50	not rep.	0	not rep.	rep., r.u.	not rep.
Malar hypoplasia	50	not rep.	0	not rep.	rep., r.u.	rep., r.u.
**Other musculoskeletal features (%)**						
Joint laxity	25	rep., r.u.	not rep.	not rep.	63	19
Vertebral anomalies	25	not rep.	rep.	rep., r.u.	rep., r.u.	20
Intervertebral disc degeneration	0	not rep.	not rep.	not rep.	not rep.	90
Contractures	25	not rep.	rep., r.u.	18	rep., r.u.	not rep.
**Craniofacial features (%)**						
Micro- or retrognathia	25	31	0	2	rep., r.u.	not rep.
Midfacial dysplasia	0	61	0	not rep.	not rep.	not rep.
**Cardiovascular features**						
Aortic dilatation / dissection	0	not rep.	not rep.	not rep.	77	58
Congenital heart disease	25	not rep.	not rep.	not rep.	not rep.	9
Varicosis	25	not rep.	not rep.	not rep.	not rep.	64
**Ocular features (%)**						
Any ocular manifestation	25	100	rep., r.u.	0	54	not rep.
**Other features (%)**						
Velvety skin or Striae	0	not rep.	not rep.	not rep.	47	67
Herniae	0	not rep.	not rep.	not rep.	10	50
Hearing loss	0	18	not rep.	22	not rep.	not rep.

Comparison of the clinical presentation in our patients with related connective tissue disorders. Clinical features are listed by syndrome/disorder and frequencies of specific features are given in percentage of clinically examined patients in the respective cohort. Note the overlap in the clinical presentation of our patients with MFS and AOS concerning seven and eight main features, respectively. MASS, Myopia, mitral valve prolapse, borderline and non-progressive aortic root dilatation, skeletal findings and striae; STL1, Stickler syndrome, type I; OSCPD, osteoarthritis with mild chondrodysplasia; MFS, Marfan syndrome; LDS3, Loeys-Dietz syndrome 3; AOS, Aneurysms-Osteoarthritis syndrome; rep., reported; r.u., rate unknown.

Several syndromic disorders are caused by pathogenic variants in the *COL2A1* gene, also referred to as type II collagenopathies^4^. These disorders differ in their specific combinations of clinical features^3,11,35^. The most frequent form of type II collagenopathies, Stickler Syndrome 1 (STL1), is characterized by a multisystem involvement including ocular manifestations (congenital myopia, vitreous, retinal abnormalities), craniofacial manifestations (midface hypoplasia, micrognathia), palatal abnormalities (cleft palate, highly arched palate), skeletal manifestations (joint hypermobility, arachnodactyly, spine abnormalities, pectus deformity), sensorineural hearing loss, and early-onset degenerative arthritis^3,9^. In comparison, a type II collagenopathy involving a single organ system is the *COL2A1*-related osteoarthritis with mild chondrodysplasia (OSCDP) that is characterized by isolated skeletal/joint involvement, i.e. OA of hips, knees, shoulders, wrists, hands, joint stiffness, spine abnormalities (irregular endplates, mild platyspondyly, anterior wedging) and manifestations of the hands (enlarged MCP, PIP and DIP joints, Heberden's nodes)^3,4^. The common clinical features in our patients (i.e. present in ≥ 75%) are tall stature, arachnodactyly, dural ectasia, kyphoscoliosis, pectus deformity, foot deformity, dolichostenomelia, and high arched palate. We compared these clinical features with known *COL2A1*-related disorders (Table 2)^3,4^. Although we identified partial clinical overlap, we could not assign the presented phenotype to a known *COL2A1*-associated syndrome. For example, arachnodactyly, scoliosis, pectus deformity, and OA are features of STL1; however, tall stature, dolichocephaly, dolichostenomelia, malar hypoplasia, feet deformitiy, and dural ectasia have not been associated with this syndrome (Table 2)^3,36^. Furthermore, only one of the four subjects presented here showed an ocular manifestation, whereas involvement of the eyes is an obligate symptom in patients with STL1^36^. Subject 1A and his mother (subject 1B) presented with OA mainly affecting the finger joints. Therefore, we have considered OSCDP as differential diagnosis, in which OA is a main feature. However, other features present in our patients such as tall stature, dural ectasia, high arched palate, dolichocephaly, malar hypoplasia, foot deformity, dolichostenomelia, and pectus deformity are not in line with this differential diagnosis (Table 2). Notably, *COL2A1* variants have been recently identified as the main monogenic cause of nonsyndromic early-onset OA^37^. Nonetheless, it remains to be determined if the joint manifestations in subjects 1A and 1B are solely attributed to the COL2A1 p.(Lys1312Asn) variant.

Taken together, in addition to the overlapping clinical manifestation with STL1, OSCDP and other type II collagenopathies, the patients of this study present with pronounced skeletal features, resembling MFS or—to a minor extent—LDS3 (Table 2). This led us to apply the revised Ghent criteria^12^, and the clinical diagnosis of a MASS-like phenotype could be established in all four patients (aortic root Z-score < 2, at least one skeletal feature [i.e. pectus deformity], and a systemic score ≥ 5). The fact that a MASS-like phenotype was considered in our clinically different patients may suggest that the diagnosis of MASS is applicable not only to *FBN1*-related disorders, but also to other hereditary disorders of connective tissue. Since our patients were young at the final examination with an average age of 33 years, we cannot exclude that aortic events may appear and evolve later in life^31^. Thus, we will validate our diagnosis of a MASS-like phenotype in a longitudinal examination of the subjects and, if need be, revise it in a Marfan syndrome-like phenotype. Despite the wide clinical spectrum of type II collagenopathies, a MASS- or Marfan syndrome-like phenotype associated with *COL2A1* variants has not been reported to date. Therefore, our findings further expand the phenotypical spectrum of *COL2A1*-related disorders.

### Genetic and molecular aspects

The VWFC repeat in the N-propeptide region of COL2A1 (Fig. 1a) is involved in protein–protein interaction with transforming growth factor $\upbeta$ (TGF$\upbeta$) and bone morphogenetic protein 2 (BMP2)^20,38^, and thereby, is able to control numerous biological processes including embryonic patterning, differentiation, and homeostasis of various tissue types^39^. Amino acid asparagine 65 (p.Asp65Asn) localizes in the so-called subdomain 1 (SD1; amino acids Asp^43^–Ile^68^) of the VWFC repeat (Fig. 1a), adjacent to Cys^64^ that links two strands of a three-stranded antiparallel $\upbeta$-sheet via a disulfide bond with Cys^55^ (Fig. S1a)^20,40^. Given the opposite orientation of Asp^65^ compared to Cys^64^ on the $\upbeta$-sheet and the only minor effects of the p.Asp65Asn molecular replacement on intramolecular contacts/clashes (Fig. S1a), it is unlikely that this change interferes with disulfide bridge formation. It has been shown, that a hydrophobic patch including the nonpolar, hydrophobic amino acids Val^46^, Ile^54^, Val^56^, Leu^63^ and Ile^67^ mediates the interaction with BMP2 (Fig. 1b)^20^. Asp^65^ that is highly conserved across different species (Fig. S1c, Table S2) borders this hydrophobic pocket. Asp^65^ is an acidic, negatively charged amino acid, and therefore, may determine the binding pocket’s physicochemical properties and substrate specificity. It can be inferred that the change of Asp^65^ by a polar, uncharged Asn very likely affects binding properties and BMP2 substrate recognition. Note that charged amino acids form salt bridges, whereas polar residues can participate in hydrogen bond formation. In analogy, the different substrate specificities of the proteases trypsin and chymotrypsin is determined in part by a single amino acid within their almost identical hydrophobic substrate-binding pocket: residue 189 is a negatively charged asparagine in trypsin and a polar serine in chymotrypsin^41^. BMP2 is an essential participant in skeletal homeostasis, and the osteogenic signal provided by BMP2 is required for inherent reparative capacity of bone^42^. Changes in BMP activity in adult bone are associated with osteoporosis, OA^43^, and in the reduced ability to heal fractures, all major health problems that increase in severity with age^42^. Thus, altered BMP signaling may underlie the clinical manifestation in subject 2.

The fibrillar collagen NC1 domain (Fig. 1a) facilitates intracellular trimerization by association of C-propeptides from three procollagen molecules followed by zipper-like folding of the triple-helical region towards the N-terminal end^44^. In addition, C-propeptides also control the assembly of collagen fibrils in the ECM by maintaining the solubility of trimers^44^. The structure model of COL2A1 C-terminal propeptide homotrimer (Fig. S4) based on highly homologous COL1A1^21^ shows that the amino acids Lys^1312^ and Ser^1338^ (and also the homologous amino acids Lys^1288^ and Ile^1314^ in COL1A1 and Lys^1291^ and Thr^1317^in COL3A1, data not shown) are not involved in the interaction between adjacent collagen alpha-chains essential for trimerization^21,45^. These amino acids are also not involved in the stabilization of intra-chain disulfide bonds relevant for protein folding^21,45^. Lys^1312^ localizes in the so-called base (Fig. S4) of the COL2A1 C-terminal propeptide in proximity to the Ca^2+^ binding loop (Figs. 1c, S2a). The base regions stabilize the procollagen trimer^21,45^. Lys^1312^ is conserved across mammalia but not across less related species (Fig. S2c, Table S2). The pathogenic variants p.Tyr1298Asn and p.Gly1305Ala, which localize near Lys^1312^ in the COL2A1 base region, have been associated with type 2 collagenopathies^11,46^. It has been suggested that these changes destabilize the base and disrupt the Ca^2+^-binding loop, respectively^45^. Similarly, our modelling data indicate that the p.(Lys1312Asn) variant compromises the shape and function of the base and/or Ca^2+^-binding loop (Fig. 1c, Fig. S2). Amino acid Ser^1338^ is located in the petal of the COL2A1 C-terminal propeptide (Fig. S4), which assures procollagen chain selectivity^21,45^. However, Ser^1338^ is not directly involved in inter-chain interactions^21^. Ser^1338^ is conserved across vertebrates but not across less related species (Fig. S3c, Table S2). The p.(Ser1338Asn) change induces marked structural alteration that mainly concerns Trp^1336^ (Fig. 1d, Fig. S3a). A pathogenic variant affecting the homologous amino acid in COL1A1 (p.Trp1312Cys) with putative negative consequences on intramolecular interactions has been identified in a patient with a lethal form of osteogenesis imperfect^45,47^. Moreover, the pathogenic variant p.Lys1313Arg in COL3A1 (homologous to COL2A1 Lys^1334^, Fig. S3a) that probably disrupts surface interactions has been shown in a patient with vascular Ehlers-Danlos syndrome^45,48^. These data demonstrate that the molecular region around Ser^1338^ is disease critical. Based on our modelling data we conclude that the p.Ser1338Asn variant rather interferes with intramolecular interactions than affect molecule surface properties (Fig. 1d, Fig. S3). The function of C-propeptides in extracellular fibril assembly is not well established. Particularly, protein interactions and signaling functions of C-propeptides need further characterization^44^. Nonetheless, it has long been known that the release of the C-terminal propeptide decreases the solubility of collagen trimers by 1000-fold, thus leading to spontaneous fibril assembly^44,49^. Bone morphogenetic protein 1 (BMP1) is the proteinase mainly responsible for the cleavage of C-propetides and it involves cofactors such as the ECM protein procollagen C-endopeptidase enhancer (PCOLCE) that binds to C-propetides^44^. Given the surface exposition of COL2A1 amino acids Lys^1312^ and Ser^1338^ (Fig. S4), it is possible that protein–protein binding properties (binding affinities) and/or the proteolytic release of COL2A1 C-propeptide is affected, which finally may result in impaired extracellular fibril assembly. Summarized, the lack of valid data for the biological functions of COL2A1 C-propeptides—be it fibril assembly, signaling properties and/or even a structural function in the ECM—makes it challenging and speculative to assess the functional consequences of amino acid changes in this region.

### Genotype–phenotype correlation

Pathogenic variants affecting the COL2A1 N-terminal propeptide region cause mild phenotypes such as STL1^4,50^. In line with this, subject 2 with the p.(Asp65Asn) change in the VWFC region also presents with mild, preferentially skeletal features. Interestingly, the *COL2A1* missense variant c.170G > A p.(Cys57Tyr) in the VWFC region has been identified in a patient with an ocular form of Stickler syndrome but no extraocular features^51^. Though we are unable to explain the respective underlying pathophysiology, the drastic phenotypic differences between the patient with p.(Cys57Tyr) and subject 2 with p.(Asp65Asn) impressively reflect the pronounced clinical variability of type II collagenopathies^4,8,11^. The phenotypic spectrum of pathogenic missense variants affecting the COL2A1 C-propeptide is highly variable with a focus on skeletal and ocular manifestations^4,8,11,52^. Factors such as type and position of the amino acid substitution or tissue specific alternative splicing may contribute to the clinical manifestation^4,8,11,52–55^. Pathogenic *COL2A1* C-propeptide variants are especially known to result in atypical, severe and lethal phenotypes^4,8,46,52,53,56–59^. However, this correlation is not perfect, because mild phenotypes such as STL1 or Czech dysplasia (MIM # 609162) have also been associated with C-terminal variants^4,11,52^. Accordingly, our two patients with the missense variants affecting the C-terminal fibrillar collagen NC1 domain presented with mild clinical manifestations. Thus, our data support the observation that pathogenic variants affecting surface-located C-propeptide residues result in mild to moderate phenotypes, whereas those that interfere with inter- or intramolecular interactions are usually associated with severe phenotypes^44,45^.

### Limitations

This study also has limitations. Intronic variants have been identified in *FBN1*-related disorders^27,60^; such variants are missed by tNGS. Thus, in cannot be ruled out that deep intronic *FBN1* variants cause the clinical manifestation in our patients. Likewise, tNGS does not capture structural variants, copy number variations or non-coding variants (*e.g.*, in promoters or enhancers), which also applies to the *FBN1* gene. Finally, only 62 genes were screened in our study; however, variants in untested genes may also underlie the clinical manifestation of the presented individuals.

## Conclusion

We report here for the first time that *COL2A1* variants are associated with a MASS-like phenotype characterized by tall stature, arachnodactyly, spine deformity, dolichostenomelia, foot deformity, arched palate, and pectus deformity. As dural ectasia and dolichocephaly have not been described in type II collagenopathies, our data expand the clinical spectrum associated with *COL2A1* variants. The delineation of known *COL2A1*-associated phenotypes and definition of new ones can be helpful in the establishment of a valid genotype–phenotype correlation. Patients suggestive for MASS-like phenotype or Marfan-like syndrome (without aortopathy) and negative for disease-relevant *FBN1* variants should be tested for sequence alterations in *COL2A1*.

### Ethics approval

This study was performed in line with the principles of the Declaration of Helsinki. Approval was granted by the Medical Chamber Hamburg (vote no. PV7038).

### Consent to participate

The patients or patients’ parents provided written informed consent for the participation in the study, clinical data and specimen collection and genetic analysis according to the Declaration of Helsinki and the national legal regulations [e.g., the German Genetic Diagnosis Act (GenDG)].

### Consent for publication

The patients or patients’ parents signed informed consent regarding publishing their data and photographs.

## Data availability

All data generated or analysed during this study are included in this published article [and its supplementary information files]. All variants and associated phenotypes have been submitted to the ClinVar database (submission name SUB9514030).

## Supplementary Information

Supplementary Information.

Supplementary Figure S1.

Supplementary Figure S2.

Supplementary Figure S3.

Supplementary Figure S4.
