# Supplementary material for: Expanding the clinical spectrum of COL2A1 related disorders by a mass like phenotype
Source: Sci Rep. 2022 Mar 16;12:4489. doi: 10.1038/s41598-022-08476-7 (PMC8927422; doi:10.1038/s41598-022-08476-7)
Supplement: Supplementary file 1 — Supplementary Information. [file 41598_2022_8476_MOESM1_ESM.docx]

**SUPPLEMENTARY INFORMATION**

**Expanding the clinical spectrum OF *COL2A1* related disorders by a MASS like phenotype**

Till Joscha Demal^1§^, Tasja Scholz^2§^, Helke Schüler^3^, Jakob Olfe^4^, Anja Fröhlich^5^, Fabian Speth^5^, Yskert von Kodolitsch^3^, Thomas S. Mir^4^, Hermann Reichenspurner^1^, Christian Kubisch^2^, Maja Hempel^2†‡^, Georg Rosenberger^2†*^

^1^Department of Cardiovascular Surgery, University Heart and Vascular Center Hamburg, Hamburg, Germany

^2^Institute of Human Genetics, University Medical Center Hamburg-Eppendorf, Hamburg, Germany

^3^Department of Cardiology, University Heart & Vascular Center Hamburg, Hamburg, Germany

^4^Pediatric Cardiology Clinic, University Heart & Vascular Center Hamburg, Hamburg, Germany

^5^Department of Paediatrics, University Medical Center Hamburg-Eppendorf, Hamburg, Germany

^§^ These authors (TJD and TS) contributed equally to this work.

^†^ These authors (MH and GR) also contributed equally to this work.

^‡^ Present address: Genetic Clinic, Institute of Human Genetics, Heidelberg University Hospital, Heidelberg, Germany

**Short title**: *COL2A1* variants cause MASS like phenotype

***CORRESPONDING AUTHOR**

**Georg Rosenberger, MSc, PhD**

ORCID 0000-0003-3625-3109

Phone +49-40-741054534

Email rosenberger@uke.de

**SUPPLEMENTARY MATERIALS AND METHODS**

**Clinical examination**

Aortic root measurements were done according to the Ghent nosology guidelines using transthoracic echocardiography in parasternal long-axis, parallel to the plane of the aortic valve, and perpendicular to the axis of blood flow. The largest correctly measured root diameter obtained from at least three measurements was corrected for age and body size and interpreted as a Z-score^1^. Echocardiographic measurement technique (internal diameter vs. leading-edge-to-leading-edge) was matched to the technique that was used to generate the normative data we compared our data with. In individuals ≤15 years, measurements were performed in systole from the anterior inside edge to the posterior inside edge and normative data from Lopez et al. were applied^2^. In patients >15 years, measurements were performed in diastole using the leading-edge-to-leading-edge method and normative data from Devereurx et al. were used^3^.

**Targeted next-generation sequencing**

Genomic DNA was extracted from peripheral blood using standard laboratory procedures. Targeted NGS (tNGS) was performed at the Institute for Human Genetics, University Medical Center Hamburg-Eppendorf, Hamburg, Germany as previously reported by our group^4^. 62 genes (**Tab. S1**) (including 40 bp of 5’ and 3’ intronic sequence for each exon) were defined as ROI. These genes are either associated with thoracic aortic aneurysm/dissection-spectrum disorders or with connective tissue disease or they have crucial functions in connective tissue homeostasis (**Tab. S1**). Enrichment of the regions of interest (ROI) was performed with the Nextera^TM^ Flex for Enrichment Kit (Illumina, San Diego, CA), according to the manufacturer’s instructions. Briefly, following the fragmentation of genomic DNA, fragmented DNA was amplified, and subject-specific (index) adapters were added by PCR. Samples from 12 subjects were combined into one single hybridization mix containing target-specific capture probes. The DNA-probe hybrids were then captured with streptavidin beads, and non-targeted DNA fragments as well as unspecific binding were removed by heated washes. Next, the captured DNA library was eluted from the beads, purified, and amplified by PCR. The concentration of each library was measured by Qubit fluorometric quantification (Life Technologies, Carlsbad, CA, USA). For the generation of clusters and subsequent sequencing of the targeted DNA samples on a flow cell, a sequencing reagent kit from Illumina was used. High-throughput NGS data were generated on an Illumina sequencing platform. ROI sequences were aligned to the human reference genome (hg19) and visualized and evaluated by using the Sequence Pilot module SeqNext (JSI Medical Systems). To determine true positive variants and eliminate sequencing artefacts we defined a minimal coverage of 20 reads, irrespective of the sequencing direction. Moreover, only variants covered by ≥10% of the reads, both in forward and reverse direction, were called.

**Variant prioritization and classification**

Filtering on synonymous, missense and nonsense variants, coding indels, and intronic alterations at exon-intron boundaries ranging from -10 to +10, variants with a minor allele frequency (MAF) according to the gnomAD database v.2.1.1^5^ higher than the predicted maximum population frequency (MPF) of respective gene mutations were excluded from further analysis. The MPF was calculated based on the respective disease prevalence, penetrance, and the genetic/allelic contribution of the respective gene/disease for all analyzed genes (**Tab. S1**) (Whiffin et al. 2017). For X-linked disorders we calculated the MPF by assuming monoallelic inheritance (MPF calculator is available at cardiodb.org/allelefrequencyapp/; accessed February 2021). Because Stickler syndrome, type 1 (STL1) is the most prevalent condition associated with pathogenic *COL2A1* variants^6,7^, STL1 with a prevalence of 1:8,250 was selected for *COL2A1* MPF calculation (Robin et al. 1993). Penetrance was set to 100%^8^, and the genetic 🞫 allelic contribution of pathogenic *COL2A1* variants was estimated to approximately 85% 🞫 10%^6,9-12^. This resulted in an MPF of 5.15E-06 for *COL2A1* variants (**Tab. S1**). Variants were classified according to ACMG/AMP classification criteria^13,14^, and further evaluated by determining their relationship with variants already reported in the database on the relationships between human variations and phenotypes (ClinVar) and the Human Gene Mutation Database (HGMD)^15,16^ and by predicted pathogenicity according to VarSome (version 10.2)^17^ and additional *in silico* prediction tools (**Tab. S2**). VUS, LPVs and PVs were reported.

**Sanger Sequencing (genotyping)**

Genotyping of *COL2A1* variants was performed by Sanger sequencing. PCR conditions and primer sequences are available on request. Amplicons were directly sequenced using the ABI BigDye Terminator Sequencing Kit (Applied Biosystems) and an automated capillary sequencer (ABI 3500; Applied Biosystems). Sequence electropherograms were analyzed using the Sequence Pilot software (JSI Medical Systems, Kippenheim, Germany).

**Molecular Modelling**

Molecular graphics were developed with UCSF ChimeraX (version 1.2)^18^. For molecular replacement and analysis, UCSF ChimeraX build-in tools were used. For all replaced amino acids, the most favorable torsion of the respective side chains as predicted by UCSF ChimeraX (structure editing tools – rotamers) is shown (**Figs. 1c**, **1d**, **S1a**, **S2a** and **S3a**). For modelling the structural environment of Asp^65^ and of the surface of the COL2A1 VWFC repeat, the crystallographic structure of COL2A1 VWFC (amino acids 29-98) (PDB ID 5NIR) was used^19^. For modelling the structural environment of Lys^1312^ and Ser^1338^ and the surface of the COL2A1 C-terminal propeptide, the crystallographic structure of COL1A1 C-terminal propeptide homo-trimer (amino acids 1218-1464) was used as template (PDB ID 5K31)^20^. The web-based service homology modelling of SWISS-Model was applied to build a COL2A1 model that is based on 68.72% sequence identity with COL1A1^21^. Covalent disulfide bridges and overlaps of atomic Van-der Waals (VDW) spheres within a 5.0-Å (Ångström) range were identified by using UCSF ChimeraX structure analysis tools. Contacts were defined as VDW overlaps ≥ -0.40 Å, and non-covalent clashes were defined as VDW overlaps ≥ 0.60 Å (as suggested by Chimera default settings). Contacts between atoms separated by four bonds or less as well as intra-residue contacts were ignored.

**Conservation analysis**

The degree of evolutionary conservation of the amino acids in the COL2A1 VWFC domain (amino acids 29-98) and in the C-terminal propeptide (amino acids 1250-1487) was analyzed using the ConSurf server (consurf.tau.ac.il)^22^, to find residues that are important for the function of the protein. 50 sequences of COL2A1 across species with maximal 95% identity between sequences and 35% minimal identity for homologs (i.e stringent default settings) were extracted from the Clean_Uniprot database, aligned, and scored for position specific conservation by the ConSurf Server^22,23^. The homolog search algorithm HMMER (E-value, 0.0001; No. of iterations, 1) was used (hmmer.org). The results were mapped onto the surfaces of the corresponding structures by using ChimeraX^18^. Full sequence alignments are available on request. Additional conservation scores are given and described in **Tab. S2**.

**SUPPLEMENTARY RESULTS**

**Variant classification**

We assigned ACMG/AMP criteria by using VarSome version 10.2 automated *in-silico* analysis^17^.

The ACMG/AMP guideline for **PM1** is: Variant is located in a mutational hot spot and/or critical and well-established functional domain (e.g., active site of an enzyme) without benign variation^14^. When using VarSome^17^, this criteria leverages the Varsome clinical variants database (varsome.com/about/resources/acmg-implementation/#clinicallyreportedvariants) to evaluate how many coding pathogenic variants are found near the variant being considered. Variant classifications are sourced from ClinVar, UniProt, MitoMap, publications linked by VarSome users and VarSome user classifications. Hot-Spot: using a region of 25 base-pairs on either side of the variant, the rule checks that there are at least 6 pathogenic variants (only using missense and inframe-indel variants), then weighs them by distance to compute a “proximity score”. The rule triggers with strength moderate or strong depending on the proximity and density of the hot-spot. Protein Domains: if the variant is within a functional domain reported by UniProt (uniprot.org), the rule tallies all the clinically reported pathogenic and benign variants (i.e. non-VUS and non-likely pathogenic/benign variants) within the domain. The rule will fire if the ratio of pathogenic to the total of pathogenic and benign variants is greater than 0.5, and that at least 1 pathogenic variant has been reported within the domain.

According to ACMG/AMP criteria, **PM2** is assigned for variants absent from controls (or at extremely low frequency if recessive) in Exome Sequencing Project, 1000 Genomes Project, or Exome Aggregation Consortium^14^. We assigned ACMG/AMP criteria by using automated *in silico* VarSome analysis^17^. This software assigns **PM2** for dominant genes if the allele count of the variant is less than 5 in the gnomAD database.

**PP2** is assigned for missense variants in a gene that has a low rate of benign missense variation and in which missense variants are a common mechanism of disease^14^. VarSome triggers this critera if the ratio of pathogenic to the total of non-VUS variants is greater than 0.51, and more than 12% of clinically reported variants are pathogenic.

**PP3** is assigned if there are multiple lines of computational evidence support a deleterious effect on the gene or gene product (conservation, evolutionary, splicing impact, etc.)^14^.

***COL2A1* c.3936G>T p.(Lys1312Asn)**

We classified *COL2A1* c.3936G>T p.(Lys1312Asn) as a LPV based on criteria **PM1**, **PM2**, **PP2** and **PP3**. **PM1**: The Fibrillar collagen NC1 protein domain (UniProt protein CO2A1_HUMAN) that contains amino acids Lys1312 and Ser1338 has 32 pathogenic variants versus 21 benign variants. This results in a pathogenicity rate of 60.4% that is higher than the threshold of 50.0%. **PM2**: The variant is present in one out of 251478 alleles from gnomAD v2.1.1 total population (gnomad.broadinstitute.org/variant/12-48368596-C-A?dataset=gnomad_r2_1). This dataset includes data from patients. On the other hand, *COL2A1* c.3936G>T p.(Lys1312Asn) is absent in the gnomAD v2.1.1 controls dataset with only samples from individuals who were not selected as a case in a case/control study of common disease (gnomad.broadinstitute.org/variant/12-48368596-C-A?dataset=gnomad_r2_1_controls) (**Tabs. 1** and **S2**). Taken together, assignment of **PM2** depends on the dataset used and, therefore, we applied the more objective VarSome approach. **PP2**: 322 out of 346 non-VUS missense variants in *COL2A1* are pathogenic = 93.1% which is more than threshold of 51.0%, and 582 out of 1,144 clinically reported variants in gene *COL2A1* are pathogenic = 50.9% which is more than threshold of 12.0%. **PP3**: Pathogenic computational verdict based on 13 pathogenic predictions from Polyphen-2, CADD, ClinPred, DANN, DEOGEN2, EIGEN, FATHMM-MKL, LIST-S2, M-CAP, MutationAssessor, MutationTaster, PrimateAI and SIFT vs 2 benign predictions from BayesDel_addAF and MVP (1 uncertain prediction from REVEL) (**Tabs. 1** and **S2**).

***COL2A1* c.193G>A p.(Asp65Asn)**

*COL2A1* c.193G>A p.(Asp65Asn) was classified as LPV based on criteria **PM1**, **PM2**, **PP2** and **PP3**. **PM1**: The VWFC protein domain (UniProt protein CO2A1_HUMAN) that contains Asp^65^ has 9 pathogenic variants and 1 benign variant. This results in a pathogenicity rate of 90.0% that is higher than the threshold of 50.0%. **PM2**: The variant is absent from gnomAD v2.1.1, dbSNP, 1000Genomes, NHLBI ESP EVS and HGMD databases (**Tabs. 1** and **S2**). It has previously been reported in the ClinVar database and classified as VUS (**Tabs. 1** and **S2**); however, the patient’s condition and the inheritance were not recorded. **PP2**: 322 out of 346 non-VUS missense variants in *COL2A1* are pathogenic = 93.1% which is more than threshold of 51.0%, and 582 out of 1,144 clinically reported variants in gene *COL2A1* are pathogenic = 50.9% which is more than threshold of 12.0%. **PP3**: Pathogenic computational verdict based on 11 pathogenic predictions from Polyphen-2, CADD, ClinPred, DANN, EIGEN, FATHMM-MKL, LIST-S2, M-CAP, MutationAssessor, MutationTaster and SIFT vs 4 benign predictions from BayesDel_addAF, DEOGEN2, MVP and PrimateAI (1 uncertain prediction from REVEL) (**Tabs. 1** and **S2**).

***COL2A1* c.4013G>A p.(Ser1338Asn)**

*COL2A1* c.4013G>A p.(Ser1338Asn) was classified as VUS based on criteria **PM1**, **PM2**, **PP2** and **BP4**. **PM1**: The Fibrillar collagen NC1 protein domain (UniProt protein CO2A1_HUMAN) that contains amino acids Lys1312 and Ser1338 has 32 pathogenic variants versus 21 benign variants. This results in a pathogenicity rate of 60.4% that is higher than the threshold of 50.0%. **PM2**: *COL2A1* c.4013G>A p.(Ser1338Asn) is absent from gnomAD v2.1.1, dbSNP, 1000Genomes, NHLBI ESP EVS and HGMD databases (**Tabs. 1** and **S2**). **PP2**: 322 out of 346 non-VUS missense variants in *COL2A1* are pathogenic = 93.1% which is more than threshold of 51.0%, and 582 out of 1,144 clinically reported variants in gene *COL2A1* are pathogenic = 50.9% which is more than threshold of 12.0%. **BP4**: Benign computational verdict based on 11 benign predictions from Polyphen-2, SIFT, REVEL, Mutation Taster, BayesDel_addAF, DEOGEN2, EIGEN, LIST-S2, MVP, MutationAssessor and PrimateAI vs 3 pathogenic predictions from FATHMM-MKL, M-CAP and MutationTaster (2 uncertain prediction from CADD and DANN) (**Tabs. 1** and **S2**).

**Concomitant variants**

In addition to *COL2A1* variants, gene panel sequencing revealed further reportable variants. In subject 1A the heterozygous variant c.4557C>G p.(Pro1519=) in the gene *DCHS1* was identified. According to ACMG/AMP guidelines it was classified as VUS^14^. The *DCHS1* variant did not co-segregate with the disorder in the family (subjects 1A and 1B). Biallelic pathogenic variants in *DCHS1* cause the autosomal recessive van Maldergem syndrome 1 (VMLDS1; *MIM* #601390). Heterozygous pathogenic variants are associated with the autosomal dominant mitral valve prolaps 2 (MVP2; *MIM* #607829). The clinical presentation of subject 1A does not support the diagnosis of one of these disorders. Moreover, VMLDS1 is caused by biallelic pathogenic variants and the *DCHS1* variant found in our patient was heterozygous and we detected no other variant in this gene.

Subject 3 carries the heterozygous variant c.12373G>A p.(Glu4125Lys) in the gene *TNXB,* which was classified as VUS accordning to ACMG/AMP guidelines^14^. *TNXB* is the disease gene for autosomal recessive Ehlers-Danlos syndrome, classic-like, 1 (EDSCLL; *MIM* #606408). We did not detect a second *TNXB* variant in this patient. Heterozygous *TNXB* variants are associated with an autosomal dominant vesicoureteral reflux 8 (VUR8; *MIM* #615963). Again, the clinical features present in subject 3 do not support the diagnosis of one of these disorders.

Taken together, with the exception of *COL2A1* variants, gene panel sequencing revealed no likely disease-relevant variants in our patients.

**SUPPLEMENTARY FIGURE LEGENDS**

**Figure S1. (a) Structural impact of the COL2A1 p.Asp65Asn amino acid change**. Ribbon representations of the VWFC repeat show amino acid 65 and surrounding residues within a radius of 5 Å (Ångström) as well as conserved cysteines that form five disulphide bridges (yellow lines) as sticks. Sidechains are coloured by element (hydrogen: white; carbon: beige; oxygen: red; nitrogen: blue; cysteine: yellow). The left model shows the structural environment of Asp^65^; VDW contacts of Asp^65^ with adjacent residues were not predicted. The right model shows the structural environment of Asn^65^ for which one VDW overlap ≥ -0.4 Å (contact, green line) but no VDW overlap ≥ 0.6 Å (clashes) was predicted. VWFC, von Willebrand factor (VWF) type C repeat. **(b) Visualisation of surface hydrophobicity and electrostatic potential of the COL2A1 VWFC domain (amino acids 29-98).** COL2A1 Asp^65^ affected in subject 2 and amino acids Val^46^, Ile^54^, Val^56^, Leu^63^, and Ile^67^ are labeled (left upper model). The right upper model shows the surface hydrophobicity after molecular replacement of Asp^65^ by asparagine. Different colours indicate the hydrophobicity properties of amino acids. The most polar and charged residues are in cyan and the most hydrophobic residues are in tan. The lower models depict the electrostatic potential of wild-type (p.Asp65, left) and mutated (p.Asn65, right) COL2A1 VWFC. The electrostatic potential ranges from negative (red) to positive (blue). Both, hydrophobicity and electrostatic potential were displayed by using the surface colouring feature of the UCSF ChimeraX tool (version 1.2). **(c) Conservation of amino acids (aa) 29-98 of the COL2A1 VWFC domain.** Conservation was determined between 50 sequences among various species by using the ConSurf server. ChimeraX was used for visualization. Amino acids affected in subject 2 described in this study (Asp^65^) and amino acids forming the hydrophobic BMP2 binding patch (Val^46^, Ile^54^, Val^56^, Leu^63^ and Ile^67^) in the VWFC domain are labeled. Conserved amino acids are highlighted in maroon and variable residues are in cyan.

**Figure S2. (a) Structural impact of the COL2A1 p.Lys1312Asn amino acid change**. Ribbon representations of the COL2A1 C-terminal propeptide show amino acid 1312 and surrounding residues within a radius of 5 Å (Ångström) as well as calcium (green sphere) binding residues as sticks. Sidechains are coloured by element (hydrogen: white; carbon: beige; oxygen: red; nitrogen: blue; cysteine: yellow). The left model shows the structural environment of Lys^1312^ with Van-der-Waals (VDW) overlaps ≥ -0.4 Å (contacts, green lines), whereas the right model depicts the structural environment of Asn^1312^ with VDW overlaps ≥ 0.6 Å (clashes, magenta lines). VDW contacts of Asn^65^ with adjacent residues were not predicted. **(b) Visualisation of surface hydrophobicity and electrostatic potential of the COL2A1 C-terminal propeptide (amino acids 1250-1487).** The models show surface hydrophobicity (upper models) and electrostatic potential (lower models) of wild-type (p.Lys1312, left models) and mutated (p.Asn1312, right models) COL2A1. Amino acid 1312 is labeled. Different colours indicate the hydrophobicity properties of amino acids with the most polar and charged residues in cyan and the most hydrophobic residues in tan (upper models). The electrostatic potential ranges from negative (red) to positive (blue) (lower models). Both, hydrophobicity and electrostatic potential were displayed by using the surface colouring feature of the UCSF ChimeraX tool (version 1.2). **(c)** **Conservation of amino acids 1250-1487 in the COL2A1 C-terminal propeptide**. Conservation was determined between 50 sequences among various species by using the ConSurf server. ChimeraX was used for visualization. Amino acids affected in subjects 1A and 1B described in this study (Lys^1312^) and amino acids Trp^1299^, which is affected by the p.Lys1312Asn change are labeled. Conserved amino acids are highlighted in maroon and variable residues are in cyan. Lys^1312^ is conserved on average, however, Trp^1299^ that is affected by the p.Lys1312Asn change (**Fig. S2a**) is conserved (structure model on the right).

**Figure S3. (a) Structural impact of the COL2A1 p.Ser1338Asn amino acid change**. Ribbon representations of the COL2A1 C-terminal propeptide show amino acid 1338 and surrounding residues within a radius of 5 Å as well as calcium (green sphere) binding residues as sticks. Sidechains are coloured by element (hydrogen: white; carbon: beige; oxygen: red; nitrogen: blue; cysteine: yellow). The left model shows the structural environment of Ser^1338^ with Van-der-Waals (VDW) overlaps ≥ -0.4 Å (contacts, green lines), whereas the right model depicts the structural environment of Asn^1338^ with VDW overlaps ≥ 0.6 Å (clashes, magenta lines). VDW contacts of Asn^1338^ with adjacent residues were not predicted. Lys^1334^ is labeled in beige, because it locates outside of the 5 Å radius. **(b) Visualisation of surface hydrophobicity and electrostatic potential of the COL2A1 C-terminal propeptide (amino acids 1250-1487).** The models show surface hydrophobicity (upper models) and electrostatic potential (lower models) of wild-type (p.Ser1338, left models) and mutated (p.Asn1338, right models) COL2A1. Amino acid 1338 is labeled. Different colours indicate the hydrophobicity properties of amino acids with the most polar and charged residues in cyan and the most hydrophobic residues in tan (upper models). The electrostatic potential ranges from negative (red) to positive (blue) (lower models). Both, hydrophobicity and electrostatic potential were displayed by using the surface colouring feature of the UCSF ChimeraX tool (version 1.2). **(c)** **Conservation of amino acids 1250-1487 in the COL2A1 C-terminal propeptide**. Conservation was determined between 50 sequences among various species by using the ConSurf server. ChimeraX was used for visualization. Amino acids affected in subject 3 described in this study (Ser^1338^) is labeled. Conserved amino acids are highlighted in maroon and variable residues are in cyan. Ser^1338^ is a variable amino acid according this stringent ConSurf analysis. On the other hand, by using other tools such as PhyloP, PhastCons and GERP and more related homologous sequences, Ser^1338^ turns out to be conserved (**Tab. S2**).

**Figure S4. Accessible surfaces of the COL2A1 C-terminal propeptide trimer (right).** The surface of chain A of the homotrimeric COL2A1 C-terminal propeptide is shown in transparent blue, the positions of COL2A1 Lys^1312^ and Ser^1338^ affected in three of our patients are indicated by red colour. The homologous amino acids in COL1A1 and COL3A1 are given. The structural motives petal, base and stalk are indicated. Chains B and C of the homotrimeric COL2A1 C-terminal propeptide are shown in green and red, respectively. Surface colouring feature of the UCSF ChimeraX tool (version 1.2) was used.

**Table S1. Prevalence and penetrance of disorders associated with the analysed 62 genes and maximum calculated population frequency (MPF) of causative variants.**

| Gene | NM-Nummer | Disease (Inheritance) | Prevalence^a^  Penetrance^b^ | Maximum genetic contribution (genetic heterogeneity)^c^ | Maximum allelic contribution (allelic heterogeneity)^d^ | Maximal Population Frequency (MPF) | References and databases |
| --- | --- | --- | --- | --- | --- | --- | --- |
| *ACTA2* | NM_001613.2 | Aortic aneurysm, familial thoracic 6 (AD) | 1:1,000  50% | 12-21% (20%) | 8% | 1.60E-05 | ^24,25^; LOVD |
|  |  | Multisystemic smooth muscle dysfunction syndrome (AD) | 1:1,000,000  unknown (est. 50%) | unknown (100%^†^) | 8% | 8.00E-08 | ORPHA:91387; LOVD |
| *ADAMTS10* | NM_030957.3 | Weill-Marchesani syndrome 1, recessive (AR) | 1:100,000  100% | 35% | 10%**^§^** | 1.87E-04 | ^26^ |
| *ADAMTS2* | NM_014244.4 | Ehlers-Danlos syndrome, dermatosparaxis type (AR) | est. 1:1,000,000  unknown (est. 50%) | unknown (100%^†^) | 33,3% | 4.67E-04 | OMIM #225410, *604539; ORPHA:1901; ClinVar; LOVD |
| *B3GALT6* | NM_080605.3 | Ehlers-Danlos syndrome spondylodysplastic type 2 (AR) | unknown (est. 1:1,000,000)  unknown (est. 50%) | only 16 cases known (100%^†^) | 9% | 1.27E-04 | ^27^; OMIM *615291, #615349; LOVD |
| *B4GALT7* | NM_007255.2 | Ehlers-Danlos syndrome, spondylodysplastic type, 1 (AR) | unknown (est. 1:1,000,000)  unknown (est. 50%) | unknown (100%^†^) | 50% | 7.07E-04 | ^27^; OMIM *604327, #130070; LOVD |
| *BGN* | NM_001711.5 | Aortic aneurysm, familial thoracic/Meester-Loeys syndrome (XL) | 1:1,000  50% | rare (1%) | 100% | 1.00E-05**^e^** | ^24,25^; OMIM *301870, #300989 |
| *CBS* | NM_000071.2 | Homocystinuria, B6-responsive and nonresponsive types (AR) | 1:200,000-1:335,000  unknown (est. 50%) | 100% | 50% | 1.58E-03 | ^28^; ORPHA:394 |
| *CHST14* | NM_130468.3 | Ehlers-Danlos syndrome, musculocontractural type 1 (AR) | unknown (est. 1:1,000,000)  unknown (est. 50%) | unknown (100%^†^) | 25% | 0.000354 | ^29,30^; OMIM #601776, *608429; ORPHA:2953; LOVD |
| *COL1A1* | NM_000088.3 | Ehlers-Danlos syndrome, classic type (AD) | 1:20,000  unknown (est. 50%) | 1% | 50% | 2.50E-07 | ^27,31^ |
|  |  | Ehlers-Danlos syndrome, arthrochalasia type 1 (AD) | unknown (est. 1:1,000,000)  unknown (est. 50%) | unknown (100%^†^) | 10%**^§^** | 1.00E-07 | ^27^; ORPHA:1899; LOVD; OMIM #130060, *120150 |
| *COL1A2* | NM_000089.3 | Ehlers-Danlos syndrome, cardiac valvular type, 2 (AR) | 1:1,000,000  unknown (est. 50%) | unknown (100%^†^) | 2%-10% | 0.000141 | ^27^; ORPHA:230851; LOVD, OMIM #225320, *120160; LOVD |
|  |  | Ehlers-Danlos syndrome, arthrochalasia type, 2 (AD) | unknown (est. 1:1,000,000)  unknown (est. 50%) | unknown (100%^†^) | 4%-10% | 1.00E-07 | ^27^; ORPHA:1899; LOVD; OMIM 617821, *120160; LOVD |
| *COL2A1* | NM_001844.4 | Stickler syndrome, type I (AD) | 1:7,500-1:9,000  100% | 80-90% | 2%-10% | 5.15E-06 | ^6,8,11,12,32^; LOVD |
|  |  | Czech dysplasia (AD) | 1:1,000,000  100% | 100% | 100% | 5.00E-07 | OMIM #609162, *120140 |
| *COL3A1* | NM_000090.3 | Aortic aneurysm, familial thoracic n.s. (AD) | 1:1,000  50% | rare (1%) | 100% | 1.00E-05 | ^24,25^ |
|  |  | Ehlers-Danlos syndrome, vascular type (AD) | 1:50,000  100% | 95% | 4% | 3.80E-07 | ^33^; LOVD |
| *COL4A1* | NM_001845.5 | Angiopathy, hereditary, with nephropathy, aneurysms, and muscle cramps (AD) | fewer than 100 families (est. 1:1,000,000)  100% | 100% | 7.5% | 4.00E-08 | ^34^; LOVD |
| *COL4A5* | NM_000495.4 | Alport syndrome 1, X-linked (XLD) | 1:50,000  unknown (est. 50%) | 85% | 10% | 1.70E-06**^e^** | ^35^; LOVD |
| *COL5A1* | NM_000093.4 | Ehlers-Danlos syndrome, classic type (AD) | 1:20,000  unknown (est. 50%) | 78% | 5% | 1.95E-06 | ^31^; LOVD |
| *COL5A2* | NM_000393.4 | Ehlers-Danlos syndrome, classic type (AD) | 1:20,000  unknown (est. 50%) | 14% | 10% | 3.50E-07 | ^31^; LOVD |
| *DCHS1* | NM_003737.3 | Mitral valve prolapse 2 (AD) | 1:40  unknown (est. 50%) | 25% | 10%**^§^** | 6.25E-04 | ^36^; OMIM #607829, *603057 |
| *EFEMP2* | NM_016938.4 | Cutis laxa, type IB (AR) | 1:4,000,000  incomplete (est. 50%) | 100% | 25% | 1.77E-04 | ^37^; ClinVar |
| *ELN* | NM_001278939.1 | Cutis laxa (AD) | rare (1:1,000,000)  incomplete (est. 50%) | unknown (100%^†^) | 10%**^§^** | 1.00E-07 | ^38^; OMIM #123700, *130160 |
| *EMILIN1* | NM_007046.3 | Aortic aneurysm, familial thoracic n.s. (AD) | 1:1,000  50% | <1%  (in one family) | 100% | 1.00E-05 | ^24,25,39^ |
| *FBLN5* | NM_006329.3 | Cutis laxa, autosomal recessive, type IA (AR) | Rare (1:1,000,000)  incomplete (est. 50%) | 100% | 10%**^§^** | 1.41E-03 | ^40^ |
| *FBN1* | NM_000138.4 | Marfan syndrome (AD) | 1:7,500  100% | >95% | 3% | 2.00E-06 | ^41,42^; LOVD |
|  |  | Aortic aneurysm, familial thoracic n.s. (AD) | 1:1,000  50% | 3% | 100% | 3.00E-05 | ^24,25^ |
| *FBN2* | NM_001999.3 | Contractural arachnodactyly, congenital (AD) | unknown (est. 1:20,000)  100% | 25%-75 | 9.7% | 2.25e-06 | ^43,44^ |
| *FKBP14* | NM_017946.3 | Ehlers-Danlos syndrome, kyphoscoliotic type, 2 (AR) | 1:100,000  unknown (est. 50%) | 100% | 70% | 3.13E-03 | ^27,45^; LOVD |
| *FLNA* | NM_001110556.1 | Otopalatodigital syndrome, type 1 (XLD) | unknown (est. 1:1,000,000)  est. 100% (in males) | 94% | 16% | 7.52E-08**^e^** | ^46^; LOVD |
|  |  | Heterotopia, periventricular, 1 (XLD) | unknown (est. 1:500,000)  est. 100% (in males) | 95% | 10%**^§^** | 9.50E-08**^e^** | ^47^ |
|  |  | Frontometaphyseal dysplasia 1 (XLR) | unknown (est. 1:1,000,000)  unknown (est. 50%) | 71% | 10%**^§^** | 1.19E-04**^e^** | ^46^ |
| *FLNC* | NM_001458.4 | Cardiomyopathy, familial restrictive 5 (AD) | unknown (est. 1:333,000)  unknown (est. 50%) | unknown (100%^†^) | 6% | 1.80E-07 | ^48^; OMIM #617047, *102565; LOVD |
| *FOXE3* | NM_012186.2 | Aortic aneurysm, familial thoracic 11 (AD) | 1:1,000  50% | 1.4% | 100% | 1.40E-05 | ^25,49^;  OMIM # 617349, *601094 |
| *GATA5* | NM_080473.4 | Congenital heart defects, multiple types, 5 (AR, AD) | 1:100 (CHD in general)  incomplete (est. 50%) | 2% | 10%**^§^** | 2.00E-05 | ^50-54^; OMIM #617912, *611496 |
| *KDR* | NM_002253.2 | Hemangioma, capillary infantil (AD, AR) | est. 1:10  unknown (est. 50%) | est. 1% | 10%**^§^** | 1.00E-04 | ^55^; OMIM #602089; *191306 |
| *LOX* | NM_002317.6 | Aortic aneurysm, familial thoracic 10 (AD) | 1:1,000  50% | 1.5% | 100% | 1.50E-05 | ^24,25^ |
| *LRP1* | NM_002332.2 | Aortic aneurysm, familial thoracic n.s. (risk factor) | 1:1,000  50% | unknown (100%^†^) | 10%**^§^** | 1.00E-04 | ^56^ |
|  |  | Keratosis pilaris atrophicans (AR) | 1:8  est. 50% | unknown (100%^†^) | 10%**^§^** | 1.00E-04 | ^57^;  OMIM #604093 |
| *LTBP2* | NM_000428.2 | Weill-Marchesani syndrome 3 (AR) | 1:100,000  100% | 4% | 20% | 1.26E-04 | ^26^; LOVD |
| *LTBP4* | NM_003573.2 | Cutis laxa, autosomal recessive, type IC (AR) | rare (est. 1:1,000,000)  unknown (est. 50%) | 100% | 15% | 1.98E-04 | ^58^; LOVD |
| *MAT2A* | NM_005911.5 | Aortic aneurysm, familial thoracic n.s. (AD) | 1:1,000  50% | 1% | 100% | 1.00E-05 | ^24,25^ |
| *MFAP5* | NM_003480.3 | Aortic aneurysm, familial thoracic 9 (AD) | 1:1,000  50% | 0.25% | 100% | 2.50E-06 | ^24,25^ |
| *MYH11* | NM_001040113.1 | Aortic aneurysm, familial thoracic 4 (AD) | 1:1,000  50% | 1% | 100% | 1.00E-05 | ^24,25^ |
| *MYLK* | NM_053025.3 | Aortic aneurysm, familial thoracic 7 (AD) | 1:1,000  50% | 1% | 100% | 1.00E-05 | ^24,25^ |
| *NOTCH1* | NM_017617.4 | Aortic valve disease 1 (AD) | 1:50-500 (1:275)  unknown (est. 50%) | 5% | 10%**^§^** | 1.82E-05 | ^59,60^; OMIM #109730, *190198 |
|  |  | Adams-Oliver syndrome 5 (AD) | 1:230,000  Incomplete (est. 50%) | 23% | 10%**^§^** | 1.00E-07 | ^61^ |
| *PLOD1* | NM_000302.3 | Ehlers-Danlos syndrome, kyphoscoliotic type, 1 (AR) | 1:100,000  100% | unknown (100%^†^) | 30% | 9.49E-04 | ^27,62^; LOVD |
| *PLOD3* | NM_001084.4 | Lysyl hydroxylase 3 deficiency (AR, risc factor) | unknown (est. 1:1,000,000)  unknown (est. 50%) | unknown (100%^†^) | 10%**^§^** | 1.41E-04 | ^63^; OMIM #612394, *603066 |
| *PRDM5* | NM_018699.3 | Brittle cornea syndrome 2 (AR) | 1:1,000,000  unknown (est. 50%) | unknown (100%^†^) | 15% | 2.12E-04 | ^27,64^; OMIM #614170, *614161; ORPHA:90354 |
| *PRKG1* | NM_006258.3 | Aortic aneurysm, familial thoracic 8 (AD) | 1:1,000  50% | 1% | 100% | 1.00E-05 | ^24,25^ |
| *SKI* | NM_003036.3 | Sphrintzen-Goldberg syndrome (AD) | rare (est. 1:1,000,000)  unknown (est. 50%) | 100% | 10% | 1.00E-07 | ^65,66^; ClinVar; OMIM #182212, *164780; LOVD |
| *SLC2A10* | NM_030777.3 | Arterial tortuosity syndrome (AR) | rare (est. 1:1,000,000)  unknown (est. 50%) | unknown (100%^†^) | 20% | 2.00E-07 | ^67^; ClinVar; OMIM *606145, #208050; LOVD |
| *SLC39A13* | NM_152264.4 | Ehlers-Danlos syndrome, spondylodysplastic type, 3 (AR) | unknown (est. 1:1,000,000)  unknown (est. 50%) | unknown (100%^†^) | 20% | 0.000283 | ^27,68^; OMIM #612350, *608735 |
| *SMAD2* | NM_005901.5 | Aortic and arterial aneurysmal disease and connective  tissue features (AD, risc factor) | unknown (est. 1:1,000,000)  unknown (est. 50%) | unknown; appr.20 cases decribed (100%^†^) | 10%**^§^** | 1.00E-07 | ^69-73^ |
|  |  | Loeys-Dietz syndrome (AD) | unknown (est. 1:100,000)  95% | 1-5% | 20% | 5.26E-08 | ^73,74^; UMD |
| *SMAD3* | NM_005902.3 | Aortic aneurysm, familial thoracic n.s. (AD) | 1:1,000  50% | 2% | 100% | 2.00E-05 | ^24,25^ |
|  |  | Loeys-Dietz syndrome 3 (AD) | unknown (est. 1:100,000)  95% | 5-10% | 20% | 1.05E-07 | ^73,74^; UMD |
| *SMAD4* | NM_005359.5 | Juvenile polyposis/hereditary hemorrhagic telangiectasia syndrome (AD) | 1:10,000  age-dependent (50%) | 1%-2% | 25% | 5.00E-07 | ^75^, LOVD |
|  |  | Myhre syndrome (AD) | rare (1:1,000,000)  100% | 100% | 25% | 2.50E-07 | ^76^; LOVD |
|  |  | Polyposis, juvenile intestinal (AD) | 1:50,000  97% | 27% | 25% | 6.96E-07 | ^77^; LOVD |
| *SOX18* | NM_018419.2 | Hypotrichosis-lymphedema-telangiectasia syndrome (AR) | unknown (est. 1:1,000,000)  unknown (est. 50%) | unknown (100%^†^) | 10%**^§^** | 0.000141 | ^78^; ORPHA:69735; OMIM #607823 |
|  |  | Hypotrichosis-lymphedema-telangiectasia-renal defect syndrome (AD) | unknown (est. 1:1,000,000)  unknown (est. 50%) | unknown (100%^†^) | 10%**^§^** | 1.00E-07 | ^78^; ORPHA:69735; OMIM #137940 |
| *TGFB2* | NM_001135599.2 | Aortic aneurysm, familial thoracic (AD) | 1:1,000  50% | 1% | 100% | 1.00E-5 | ^24,25^ |
|  |  | Loeys-Dietz syndrome 4 (AD) | unknown (est. 1:100,000)  95% | 5%-10% | 20% | 1.05E-07 | ^73,74^; UMD |
| *TGFB3* | NM_003239.3 | Aortic aneurysm, familial thoracic (AD) | 1:1,000  50% | rare (est. 1%) | 100% | 1.00E-05 | ^24,25^ |
|  |  | Loeys-Dietz syndrome 5 (AD) | unknown (est. 1:100,000)  95% | 1%-5% | 20% | 5.26E-08 | ^73,74^; UMD |
| *TGFBR1* | NM_004612.3 | Loeys-Dietz syndrome 1 (AD) | unknown (est. 1:100,000)  95% | 20%-25% | 20% | 2.63E-07 | ^73,74^; UMD |
|  |  | Aortic aneurysm, familial thoracic n.s. (AD) | 1:1,000  50% | 3% | 100% | 3.00E-05 | ^24,25^ |
| *TGFBR2* | NM_001024847.2 | Loeys-Dietz syndrome 2 (AD) | unknown (est. 1:100,000)  95% | 55%-60% | 10-20% | 6.32E-07 | ^73,74,79^; LOVD; UMD |
|  |  | Aortic aneurysm, familial thoracic n.s. (AD) | 1:1,000  50% | 5% | 10% | 5.00E-06 | ^24,25^; LOVD |
| *TNXB* | NM_019105.6 | Ehlers-Danlos syndrome, classic-like, 1 (AR, AD; n.s.) | unknown (est. 1:1,000,000)  unknown (est. 50%) | unknown (100%^†^) | 17% | 2.40E-04 | ^27^; OMIM #606408, *600985; ORPHA:230839; LOVD |
| *ULK4* | NM_017886.3 | Aortic aneurysm, familial thoracic (n.s., risc factor) | 1:1,000  50% | rare (est. 1%) | 100% | 1.00E-05 | ^56^ |
| *ZNF469* | NM_001127464.2 | Brittle cornea syndrome 1 (AR) | 1:1,000,000  unknown (est. 50%) | unknown (100%^†^) | 15% | 2.12E-04 | ^27,64^; OMIM #229200, *612078; ORPHA:90354 |
| *GENE 57* | n.g. | n.g. | n.g. | n.g. | n.g. | 1.00E-04 | candidate disease gene |
| *GENE 58* | n.g. | n.g. | n.g. | n.g. | n.g. | 1.00E-04 | candidate disease gene |
| *GENE 59* | n.g. | n.g. | n.g. | n.g. | n.g. | 1.00E-04 | candidate disease gene |
| *GENE 60* | n.g. | n.g. | n.g. | n.g. | n.g. | 1.00E-04 | candidate disease gene |
| *GENE 61* | n.g. | n.g. | n.g. | n.g. | n.g. | 1.00E-04 | candidate disease gene |
| *GENE 62* | n.g. | n.g. | n.g. | n.g. | n.g. | 1.00E-04 | candidate disease gene |

**Table S1. Prevalence and penetrance of disorders associated with the analyzed 62 genes and maximum calculated population frequency (MPF) of causative variants.** For at least one condition per gene the respective data are given. MPF calculator is available at cardiodb.org/allelefrequencyapp/; accessed February 2021. For genes associated with more than one disorder (e.g. *ACTA2*, *COL2A1*) two or three representative disorders are given, and we used the highest MPF for variant prioritization (which is usually corresponding to the most prevalent condition). Stickler syndrome type I was selected for *COL2A1*, because it is the most prevalent condition associated with pathogenic *COL2A1* variants. Genes 57-62 are candidate disease genes, which we have identified in families with TAAD or connective tissue disorder. Currently, we investigate the role of these candidate disease genes in the pathogenesis of a.m. disorders; thus, these genes are subject of ongoing research and their names are not given (n.g.) in this table.

**^a^**, For disorders with unknown prevalence, information about similar disorders was adopted.

**^b^**, For disorders with unknown, incomplete or age-dependent penetrance, a value of 50% was estimated.

**^c^**, Maximum genetic contribution: proportion of families with pathogenic variant in this gene^80^. If genetic heterogeneity of a disorder is not well characterized, we assume maximal genetic contribution (i.e. 100%; indicated by †), so that the disease is modeled as attributable to one gene.

^d^, Maximum allelic contribution: for disorders with well characterized allelic contribution we used various databases to estimate the maximum allelic contribution at the upper bound of a 95% confidence interval (CI)^80^. Where no mutation database exists, we used what is known about similar disorders such as Marfan syndrome, vascular Ehlers-Danlos syndrome, etc. to estimate the maximum allelic contribution: no single variant causes more than 10% of cases (which is a very conservative estimation; indicated by §). If allelic heterogeneity of a disorder is not well characterized, we assume maximal allelic contribution (i.e. 100%), so that the contribution of each gene is modeled as attributable to one allele, and the maximum allelic contribution is substituted by the maximum genetic contribution (i.e. the maximum proportion of the disease attributable to a single gene)^80^. For example, familial thoracic aortic aneurysm (also known as non-syndromic TAAD) is caused by variants in the *MYLK* gene in approximately 1% of cases. Taking 0.01 as our maximum genetic contribution, a minimal allelic heterogeneity (i.e. maximal allelic contribution, 100%) and a population prevalence of 1:1,000, we derive a maximum tolerated population frequency of 0.0002).

**^e^**, For X-linked disorders we calculated the MPF by assuming monoallelic inheritance.

ORPHA, Orphanet, An online database of rare diseases and orphan drugs. Copyright, INSERM 1997. Available at www.orpha.net. Accessed February.

OMIM, Online Mendelian Inheritance in Man, An Online Catalog of Human Genes and Genetic Disorders, Available at www.omim.org/. Accessed February 2021.

LOVD, Leiden Open-source Variation Database^9^, Available at www.lovd.nl/. Accessed February 2021.

ClinVar, database on the relationships between human variations and phenotypes^15^. Available at www.ncbi.nlm.nih.gov/clinvar/. Accessed February 2021.

UMD, The UMD mutations database. Available at www.umd.be. Accessed February 2021.

AD, autosomal dominant; AR, autosomal recessive; XLD, X-linked dominant; XLR, X-linked recessive; XL, X-linked; n.s., not solved; est., estimated; n.a., not applicable; n.g., not given.

**Table S2. Details on variant frequencies, pathogenicity predictions, conservation of affected amino acids and variant classification**

| **PATIENT** | **Subjects 1A and 1B** | **Subject 2** | **Subject 3** |
| --- | --- | --- | --- |
| ***COL2A1* (NM_001844.5) variant**  **(c. notation, p. notation)** | c.3936G>T (het)  p.(Lys1312Asn) | c.193G>A (het)  p.(Asp65Asn) | c.4013G>A (het)  p.(Ser1338Asn) |
| **FREQUENCIES** | | | |
| **gnomAD v2.1.1 total population (AF) ^*^**  **(allele count/allele number/hom)** | 0.000003976  (1/251478/0) | 0  (0/280928/0) | 0  (0/251496/0) |
| **gnomAD v2.1.1 controls (AF) ^†^**  **(allele count/allele number/hom)** | 0  (0/109400/0) | 0  (0/119504/0) | 0  (0/109408/0) |
| **dbSNP ^‡^** | rs745788222 | N.l. | N.l. |
| **1000Genomes ^§^** | N.l. | N.l. | N.l. |
| **NHLBI ESP EVS ^∥^** | N.l. | N.l. | N.l. |
| **HGMD ^¶^** | N.l. | N.l. | N.l. |
| **ClinVar** ^**^ | N.l. | VUS **^††^** | N.l. |
| ***IN SILICO* PATHOGENOCITY PREDICTIONS** | | | |
| **Polyphen-2** (score/prediction) | 1.0/prob. damaging | 0.99/prob. damaging | 0.157/benign |
| **SIFT** (score/prediction) | 0.003/damaging | 0.002/damaging | 0.329/tolerated |
| **CADD** (score) | 24.8 | 28.1 | 20.5 |
| **REVEL** (score/prediction) | 0.613/uncertain | 0.551/uncertain | 0.125/benign |
| **Mutation Taster** (prediction) | Deleterious | Deleterious | Benign |
| **M-CAP** (score/prediction) | 0.148/damaging | 0.0965/damaging | 0.032/damaging |
| **ClinPred** (score/prediction) | 0.992/pathogenic | 0.964/pathogenic | 0.442/uncertain |
| **Σ VarSome pathogenicity predictions *(damaging/uncertain/tolerated)*** | 13/1/2 | 11/1/4 | 3/2/11 |
| **CONSERVATION scores of amino acids LYS^1312^, Asp^65^ And Ser^1338^ ^‡‡^** | | | |
| **PhyloP17way primate**  **(**conservation score) | 0.5989 | 0.5989 | 0.5989 |
| **PhyloP30way mammalian**  **(**conservation score) | 0.1379 | 1.026 | 1.026 |
| **PhyloP100way vertebrate**  **(**conservation score) | -0.023 | 7.905 | 0.186 |
| **PhastCons17way primate**  **(**probabilities of negative selection) | 0.999 | 0.999 | 0.999 |
| **PhastCons30way mammalian**  **(**probabilities of negative selection) | 1.0 | 1.0 | 1.0 |
| **PhastCons100way vertebrate**  **(**probabilities of negative selection) | 0.7799 | 1.0 | 0.99 |
| **GERP**  (rejected substitutions score) | 1.8799 | 4.5599 | 4.86 |
| **SEGREGATION** | | | |
| **Family members w/ the variant** | 2 affected | 1 affected | 1 affected  1 (clin. not exam.) |
| **Family members w/o the variant** | 2 unaffacted | None tested | 1 (clin. not exam.) |
| **CLASSIFICATION** | | | |
| **ACMG/AMP classification**  ***(Criteria)*** | LPV  *(PM1, PM2, PP2, PP3)* | LPV  *(PM1, PM2, PP2, PP3)* | VUS  *(PM1, PM2, PP2, BP4)* |

**Table S2. Details on variant frequencies, pathogenicity predictions, conservation of affected amino acids and variant classification.** Details are listed by variant. Nucleotide numbering uses +1 as the A of the ATG translation initiation codon in the reference sequence, with the initiation codon as codon 1.

Variants were classified as recommended by ACMG/AMP: VUS, variant of uncertain significance or with conflicting interpretations of pathogenicity; LPV, likely pathogenic variant. het, heterozygous; N.l., not listed; clin. not exam., clinically not examined.

**^*^**, Total gnomAD dataset (gnomad.broadinstitute.org/variant/12-48368596-C-A?dataset=gnomad_r2_1). Allele frequency (AF), total number of alleles/total number of analysed alleles/number of homozygous carriers (allele count/allele number/hom) are given.

**^†^**, The gnomAD v2.1.1 controls dataset includes only samples from individuals who were not selected as a case in a case/control study of common disease (gnomad.broadinstitute.org/variant/12-48368596-C. Allele frequency (AF), total number of alleles/total number of analysed alleles/number of homozygous carriers (allele count/allele number/hom) are given.

^‡^, The Single Nucleotide Polymorphism database (dbSNP; ncbi.nlm.nih.gov/projects/SNP)

^§^, Deep Catalog of Human Genetic Variation (internationalgenome.org)

^∥^, NHLBI Exome Sequencing Project (ESP) Exome Variant Server, evs.gs.washington.edu/EVS/

^¶^, HGMD, Human Gene Mutation Database.

^**^, ClinVar, database on the relationships between human variations and phenotypes.^15^ Available at www.ncbi.nlm.nih.gov/clinvar/. Accessed February 2021.

**^††^**, variant c.193G>A p.(Asp65Asn) has previously been reported in the ClinVar database and classified as VUS. The patient’s condition and the inheritance were not recorded.

**^‡‡^**, PhyloP and PhastCons are both methods to determine the grade of conservation of genomic regions using multiple alignments, given a phylogenetic tree and they are based on a statistical model of sequence evolution called a phylogenetic hidden Markov model (phylo-HMM)^81,82^. PhyloP17way scores are based on multiple alignments of 16 primate genome sequences to the human genome, PhyloP30way scores are based on multiple alignments of 29 mammalian genome sequences to the human genome and PhyloP100way scores are based on multiple alignments of 99 vertebrate genome sequences to the human genome. The greater the score, the more conserved the site. Positive scores, measure conservation, which is slower evolution than expected, at sites that are predicted to be conserved. Negative scores, measure acceleration, which is faster evolution than expected, at sites that are predicted to be fast-evolving. PhastCons17way scores are based on 17 primate genomes (including human), PhastCons30way scores are based on 30 mammalian genomes (including human) and PhastCons100way scores are based on 100 vertebrate genomes (including human). The phastCons scores represent probabilities of negative selection and range between 0 and 1. Genomic Evolutionary Rate Profiling (GERP) is a conservation score calculated by quantifying substitution deficits across multiple alignments of orthologues using the genomes of 35 mammals. It ranges from -12.3 to 6.17, with 6.17 being the most conserved. The score quantifies position-specific constraint in terms of rejected substitutions (RS) by estimating the actual number of substitutions at that site and subtracting it from the number expected assuming neutrality. In practice, a RS score threshold of 2 provides high sensitivity while still strongly enriching for truly constrained sites.

**REFERENCES**

1 Loeys, B. L. *et al.* The revised Ghent nosology for the Marfan syndrome. *J Med Genet* **47**, 476-485, doi:10.1136/jmg.2009.072785 (2010).

2 Lopez, L. *et al.* Relationship of Echocardiographic Z Scores Adjusted for Body Surface Area to Age, Sex, Race, and Ethnicity: The Pediatric Heart Network Normal Echocardiogram Database. *Circ Cardiovasc Imaging* **10**, doi:10.1161/CIRCIMAGING.117.006979 (2017).

3 Devereux, R. B. *et al.* Normal limits in relation to age, body size and gender of two-dimensional echocardiographic aortic root dimensions in persons >/=15 years of age. *Am J Cardiol* **110**, 1189-1194, doi:10.1016/j.amjcard.2012.05.063 (2012).

4 Renner, S. *et al.* Next-generation sequencing of 32 genes associated with hereditary aortopathies and related disorders of connective tissue in a cohort of 199 patients. *Genet Med* **21**, 1832-1841, doi:10.1038/s41436-019-0435-z (2019).

5 Karczewski, K. J. *et al.* The mutational constraint spectrum quantified from variation in 141,456 humans. *Nature* **581**, 434-443, doi:10.1038/s41586-020-2308-7 (2020).

6 Barat-Houari, M. *et al.* The expanding spectrum of COL2A1 gene variants IN 136 patients with a skeletal dysplasia phenotype. *Eur J Hum Genet* **24**, 992-1000, doi:10.1038/ejhg.2015.250 (2016).

7 Deng, H., Huang, X. & Yuan, L. Molecular genetics of the COL2A1-related disorders. *Mutat Res Rev Mutat Res* **768**, 1-13, doi:10.1016/j.mrrev.2016.02.003 (2016).

8 Robin, N. H., Moran, R. T. & Ala-Kokko, L. in *GeneReviews((R))* (eds M. P. Adam *et al.*) (1993).

9 Fokkema, I. F. *et al.* LOVD v.2.0: the next generation in gene variant databases. *Hum Mutat* **32**, 557-563, doi:10.1002/humu.21438 (2011).

10 Hoornaert, K. P. *et al.* Stickler syndrome caused by COL2A1 mutations: genotype-phenotype correlation in a series of 100 patients. *Eur J Hum Genet* **18**, 872-880, doi:10.1038/ejhg.2010.23 (2010).

11 Terhal, P. A. *et al.* A study of the clinical and radiological features in a cohort of 93 patients with a COL2A1 mutation causing spondyloepiphyseal dysplasia congenita or a related phenotype. *American journal of medical genetics. Part A* **167A**, 461-475, doi:10.1002/ajmg.a.36922 (2015).

12 Wang, D. D. *et al.* Mutation Spectrum of Stickler Syndrome Type I and Genotype-phenotype Analysis in East Asian Population: a systematic review. *BMC Med Genet* **21**, 27, doi:10.1186/s12881-020-0963-z (2020).

13 Biesecker, L. G. & Harrison, S. M. The ACMG/AMP reputable source criteria for the interpretation of sequence variants. *Genet Med*, doi:10.1038/gim.2018.42 (2018).

14 Richards, S. *et al.* Standards and guidelines for the interpretation of sequence variants: a joint consensus recommendation of the American College of Medical Genetics and Genomics and the Association for Molecular Pathology. *Genet Med* **17**, 405-424, doi:10.1038/gim.2015.30 (2015).

15 Landrum, M. J. *et al.* ClinVar: improving access to variant interpretations and supporting evidence. *Nucleic acids research* **46**, D1062-D1067, doi:10.1093/nar/gkx1153 (2018).

16 Stenson, P. D. *et al.* Human Gene Mutation Database (HGMD): 2003 update. *Hum Mutat* **21**, 577-581, doi:10.1002/humu.10212 (2003).

17 Kopanos, C. *et al.* VarSome: the human genomic variant search engine. *Bioinformatics* **35**, 1978-1980, doi:10.1093/bioinformatics/bty897 (2019).

18 Pettersen, E. F. *et al.* UCSF Chimera--a visualization system for exploratory research and analysis. *J Comput Chem* **25**, 1605-1612, doi:10.1002/jcc.20084 (2004).

19 Xu, E. R., Blythe, E. E., Fischer, G. & Hyvonen, M. Structural analyses of von Willebrand factor C domains of collagen 2A and CCN3 reveal an alternative mode of binding to bone morphogenetic protein-2. *J Biol Chem* **292**, 12516-12527, doi:10.1074/jbc.M117.788992 (2017).

20 Sharma, U. *et al.* Structural basis of homo- and heterotrimerization of collagen I. *Nat Commun* **8**, 14671, doi:10.1038/ncomms14671 (2017).

21 Waterhouse, A. *et al.* SWISS-MODEL: homology modelling of protein structures and complexes. *Nucleic acids research* **46**, W296-W303, doi:10.1093/nar/gky427 (2018).

22 Ashkenazy, H. *et al.* ConSurf 2016: an improved methodology to estimate and visualize evolutionary conservation in macromolecules. *Nucleic acids research* **44**, W344-350, doi:10.1093/nar/gkw408 (2016).

23 Landau, M. *et al.* ConSurf 2005: the projection of evolutionary conservation scores of residues on protein structures. *Nucleic acids research* **33**, W299-302, doi:10.1093/nar/gki370 (2005).

24 Verstraeten, A., Luyckx, I. & Loeys, B. Aetiology and management of hereditary aortopathy. *Nature reviews. Cardiology* **14**, 197-208, doi:10.1038/nrcardio.2016.211 (2017).

25 Milewicz, D. M. & Regalado, E. in *GeneReviews((R))* (eds M. P. Adam *et al.*) (1993).

26 Marzin, P., Cormier-Daire, V. & Tsilou, E. in *GeneReviews((R))* (eds M. P. Adam *et al.*) (1993).

27 Brady, A. F. *et al.* The Ehlers-Danlos syndromes, rare types. *Am J Med Genet C Semin Med Genet* **175**, 70-115, doi:10.1002/ajmg.c.31550 (2017).

28 Sacharow, S. J., Picker, J. D. & Levy, H. L. in *GeneReviews((R))* (eds M. P. Adam *et al.*) (1993).

29 Malfait, F. *et al.* Musculocontractural Ehlers-Danlos Syndrome (former EDS type VIB) and adducted thumb clubfoot syndrome (ATCS) represent a single clinical entity caused by mutations in the dermatan-4-sulfotransferase 1 encoding CHST14 gene. *Hum Mutat* **31**, 1233-1239, doi:10.1002/humu.21355 (2010).

30 Janecke, A. R. *et al.* The phenotype of the musculocontractural type of Ehlers-Danlos syndrome due to CHST14 mutations. *American journal of medical genetics. Part A* **170A**, 103-115, doi:10.1002/ajmg.a.37383 (2016).

31 Malfait, F., Wenstrup, R. & De Paepe, A. in *GeneReviews((R))* (eds M. P. Adam *et al.*) (1993).

32 Gregersen, P. A. & Savarirayan, R. in *GeneReviews((R))* (eds M. P. Adam *et al.*) (1993).

33 Byers, P. H. in *GeneReviews((R))* (eds M. P. Adam *et al.*) (1993).

34 Plaisier, E. & Ronco, P. in *GeneReviews((R))* (eds M. P. Adam *et al.*) (1993).

35 Kashtan, C. E. in *GeneReviews((R))* (eds M. P. Adam *et al.*) (1993).

36 Clemenceau, A. *et al.* Deleterious variants in DCHS1 are prevalent in sporadic cases of mitral valve prolapse. *Mol Genet Genomic Med* **6**, 114-120, doi:10.1002/mgg3.347 (2018).

37 Loeys, B., De Paepe, A. & Urban, Z. in *GeneReviews((R))* (eds M. P. Adam *et al.*) (1993).

38 Duz, M. B. *et al.* A novel case of autosomal dominant cutis laxa in a consanguineous family: report and literature review. *Clin Dysmorphol* **26**, 142-147, doi:10.1097/MCD.0000000000000179 (2017).

39 Capuano, A. *et al.* Diagnostic Exome Sequencing Identifies a Novel Gene, EMILIN1, Associated with Autosomal-Dominant Hereditary Connective Tissue Disease. *Hum Mutat* **37**, 84-97, doi:10.1002/humu.22920 (2016).

40 Van Maldergem, L. & Loeys, B. in *GeneReviews((R))* (eds M. P. Adam *et al.*) (1993).

41 Collod-Beroud, G. *et al.* Update of the UMD-FBN1 mutation database and creation of an FBN1 polymorphism database. *Hum Mutat* **22**, 199-208, doi:10.1002/humu.10249 (2003).

42 Dietz, H. in *GeneReviews((R))* (eds M. P. Adam *et al.*) (Copyright, University of Washington, Seattle (WA), 1997–2012. Available at <http://www.genetests.org>. Accessed July, 2012., 1993).

43 Frederic, M. Y. *et al.* The FBN2 gene: new mutations, locus-specific database (Universal Mutation Database FBN2), and genotype-phenotype correlations. *Hum Mutat* **30**, 181-190, doi:10.1002/humu.20794 (2009).

44 Callewaert, B. in *GeneReviews((R))* (eds M. P. Adam *et al.*) (1993).

45 Giunta, C., Rohrbach, M., Fauth, C. & Baumann, M. in *GeneReviews((R))* (eds M. P. Adam *et al.*) (1993).

46 Robertson, S. in *GeneReviews((R))* (eds M. P. Adam *et al.*) (1993).

47 Chen, M. H. & Walsh, C. A. in *GeneReviews((R))* (eds M. P. Adam *et al.*) (1993).

48 Muchtar, E., Blauwet, L. A. & Gertz, M. A. Restrictive Cardiomyopathy: Genetics, Pathogenesis, Clinical Manifestations, Diagnosis, and Therapy. *Circulation research* **121**, 819-837, doi:10.1161/CIRCRESAHA.117.310982 (2017).

49 Kuang, S. Q. *et al.* FOXE3 mutations predispose to thoracic aortic aneurysms and dissections. *The Journal of clinical investigation* **126**, 948-961, doi:10.1172/JCI83778 (2016).

50 Shi, L. M. *et al.* GATA5 loss-of-function mutations associated with congenital bicuspid aortic valve. *Int J Mol Med* **33**, 1219-1226, doi:10.3892/ijmm.2014.1700 (2014).

51 Gu, J. Y., Xu, J. H., Yu, H. & Yang, Y. Q. Novel GATA5 loss-of-function mutations underlie familial atrial fibrillation. *Clinics (Sao Paulo)* **67**, 1393-1399 (2012).

52 van der Linde, D. *et al.* Birth prevalence of congenital heart disease worldwide: a systematic review and meta-analysis. *J Am Coll Cardiol* **58**, 2241-2247, doi:10.1016/j.jacc.2011.08.025 (2011).

53 Wei, D. *et al.* GATA5 loss-of-function mutation responsible for the congenital ventriculoseptal defect. *Pediatr Cardiol* **34**, 504-511, doi:10.1007/s00246-012-0482-6 (2013).

54 Yang, Y. Q. *et al.* Mutational spectrum of the GATA5 gene associated with familial atrial fibrillation. *Int J Cardiol* **157**, 305-307, doi:10.1016/j.ijcard.2012.03.132 (2012).

55 Richter, G. T. & Friedman, A. B. Hemangiomas and vascular malformations: current theory and management. *Int J Pediatr* **2012**, 645678, doi:10.1155/2012/645678 (2012).

56 Guo, D. C. *et al.* Genetic Variants in LRP1 and ULK4 Are Associated with Acute Aortic Dissections. *Am J Hum Genet* **99**, 762-769, doi:10.1016/j.ajhg.2016.06.034 (2016).

57 Inanir, I. *et al.* Prevalence of skin conditions in primary school children in Turkey: differences based on socioeconomic factors. *Pediatr Dermatol* **19**, 307-311, doi:10.1046/j.1525-1470.2002.00087.x (2002).

58 Callewaert, B. L. & Urban, Z. in *GeneReviews((R))* (eds M. P. Adam *et al.*) (1993).

59 Mordi, I. & Tzemos, N. Bicuspid aortic valve disease: a comprehensive review. *Cardiol Res Pract* **2012**, 196037, doi:10.1155/2012/196037 (2012).

60 Meester, J. A. N. *et al.* Overlapping but distinct roles for NOTCH receptors in human cardiovascular disease. *Clinical genetics*, doi:10.1111/cge.13382 (2018).

61 Lehman, A., Wuyts, W. & Patel, M. S. in *GeneReviews((R))* (eds M. P. Adam *et al.*) (1993).

62 Yeowell, H. N., Walker, L. C., Farmer, B., Heikkinen, J. & Myllyla, R. Mutational analysis of the lysyl hydroxylase 1 gene (PLOD) in six unrelated patients with Ehlers-Danlos syndrome type VI: prenatal exclusion of this disorder in one family. *Hum Mutat* **16**, 90, doi:10.1002/1098-1004(200007)16:1<90::AID-HUMU19>3.0.CO;2-U (2000).

63 Salo, A. M. *et al.* A connective tissue disorder caused by mutations of the lysyl hydroxylase 3 gene. *Am J Hum Genet* **83**, 495-503, doi:10.1016/j.ajhg.2008.09.004 (2008).

64 Dhooge, T. *et al.* More than meets the eye: Expanding and reviewing the clinical and mutational spectrum of brittle cornea syndrome. *Hum Mutat* **42**, 711-730, doi:10.1002/humu.24199 (2021).

65 Greally, M. T. in *GeneReviews((R))* (eds M. P. Adam *et al.*) (Copyright, University of Washington, Seattle (WA), 1997–2012. Available at <http://www.genetests.org>. Accessed July, 2012., 1993).

66 Schepers, D. *et al.* The SMAD-binding domain of SKI: a hotspot for de novo mutations causing Shprintzen-Goldberg syndrome. *Eur J Hum Genet* **23**, 224-228, doi:10.1038/ejhg.2014.61 (2015).

67 Callewaert, B., De Paepe, A. & Coucke, P. in *GeneReviews((R))* (eds M. P. Adam *et al.*) (1993).

68 Giunta, C. *et al.* Spondylocheiro dysplastic form of the Ehlers-Danlos syndrome--an autosomal-recessive entity caused by mutations in the zinc transporter gene SLC39A13. *Am J Hum Genet* **82**, 1290-1305, doi:10.1016/j.ajhg.2008.05.001 (2008).

69 Micha, D. *et al.* SMAD2 Mutations Are Associated with Arterial Aneurysms and Dissections. *Hum Mutat* **36**, 1145-1149, doi:10.1002/humu.22854 (2015).

70 Granadillo, J. L. *et al.* Variable cardiovascular phenotypes associated with SMAD2 pathogenic variants. *Hum Mutat* **39**, 1875-1884, doi:10.1002/humu.23627 (2018).

71 Cannaerts, E. *et al.* Novel pathogenic SMAD2 variants in five families with arterial aneurysm and dissection: further delineation of the phenotype. *J Med Genet* **56**, 220-227, doi:10.1136/jmedgenet-2018-105304 (2019).

72 Zhang, W. *et al.* Exome sequencing identified a novel SMAD2 mutation in a Chinese family with early onset aortic aneurysms. *Clin Chim Acta* **468**, 211-214, doi:10.1016/j.cca.2017.03.007 (2017).

73 Loeys, B. L. & Dietz, H. C. in *GeneReviews((R))* (eds M. P. Adam *et al.*) (1993).

74 Loughborough, W. W. *et al.* Cardiovascular Manifestations and Complications of Loeys-Dietz Syndrome: CT and MR Imaging Findings. *Radiographics* **38**, 275-286, doi:10.1148/rg.2018170120 (2018).

75 McDonald, J. & Pyeritz, R. E. in *GeneReviews((R))* (eds M. P. Adam *et al.*) (1993).

76 Starr, L. J., Lindor, N. M. & Lin, A. E. in *GeneReviews((R))* (eds M. P. Adam *et al.*) (1993).

77 Larsen Haidle, J. & Howe, J. R. in *GeneReviews((R))* (eds M. P. Adam *et al.*) (1993).

78 Wunnemann, F. *et al.* Aortic Dilatation Associated With a De Novo Mutation in the SOX18 Gene: Expanding the Clinical Spectrum of Hypotrichosis-Lymphedema-Telangiectasia Syndrome. *Can J Cardiol* **32**, 135 e131-137, doi:10.1016/j.cjca.2015.04.004 (2016).

79 Frederic, M. Y. *et al.* A new locus-specific database (LSDB) for mutations in the TGFBR2 gene: UMD-TGFBR2. *Hum Mutat* **29**, 33-38, doi:10.1002/humu.20602 (2008).

80 Whiffin, N. *et al.* Using high-resolution variant frequencies to empower clinical genome interpretation. *Genet Med* **19**, 1151-1158, doi:10.1038/gim.2017.26 (2017).

81 Siepel, A. *et al.* Evolutionarily conserved elements in vertebrate, insect, worm, and yeast genomes. *Genome research* **15**, 1034-1050, doi:10.1101/gr.3715005 (2005).

82 Pollard, K. S., Hubisz, M. J., Rosenbloom, K. R. & Siepel, A. Detection of nonneutral substitution rates on mammalian phylogenies. *Genome research* **20**, 110-121, doi:10.1101/gr.097857.109 (2010).
