# Supplementary figures and images for: Expanding the clinical spectrum of COL2A1 related disorders by a mass like phenotype
Source: Sci Rep. 2022 Mar 16;12:4489. doi: 10.1038/s41598-022-08476-7 (PMC8927422; doi:10.1038/s41598-022-08476-7)

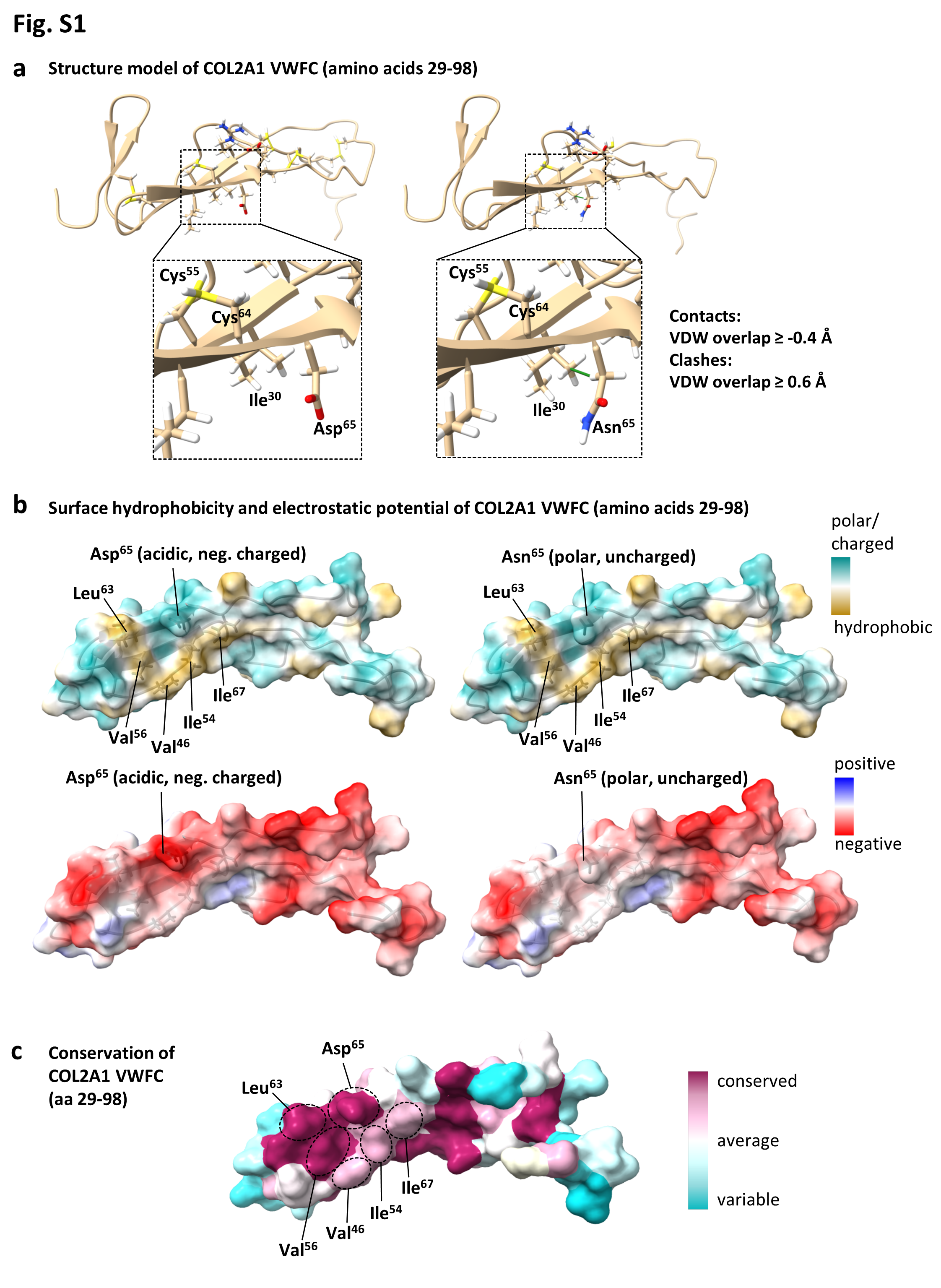

Supplement: Supplementary file 2 — Supplementary Figure S1. [file 41598_2022_8476_MOESM2_ESM.tif]

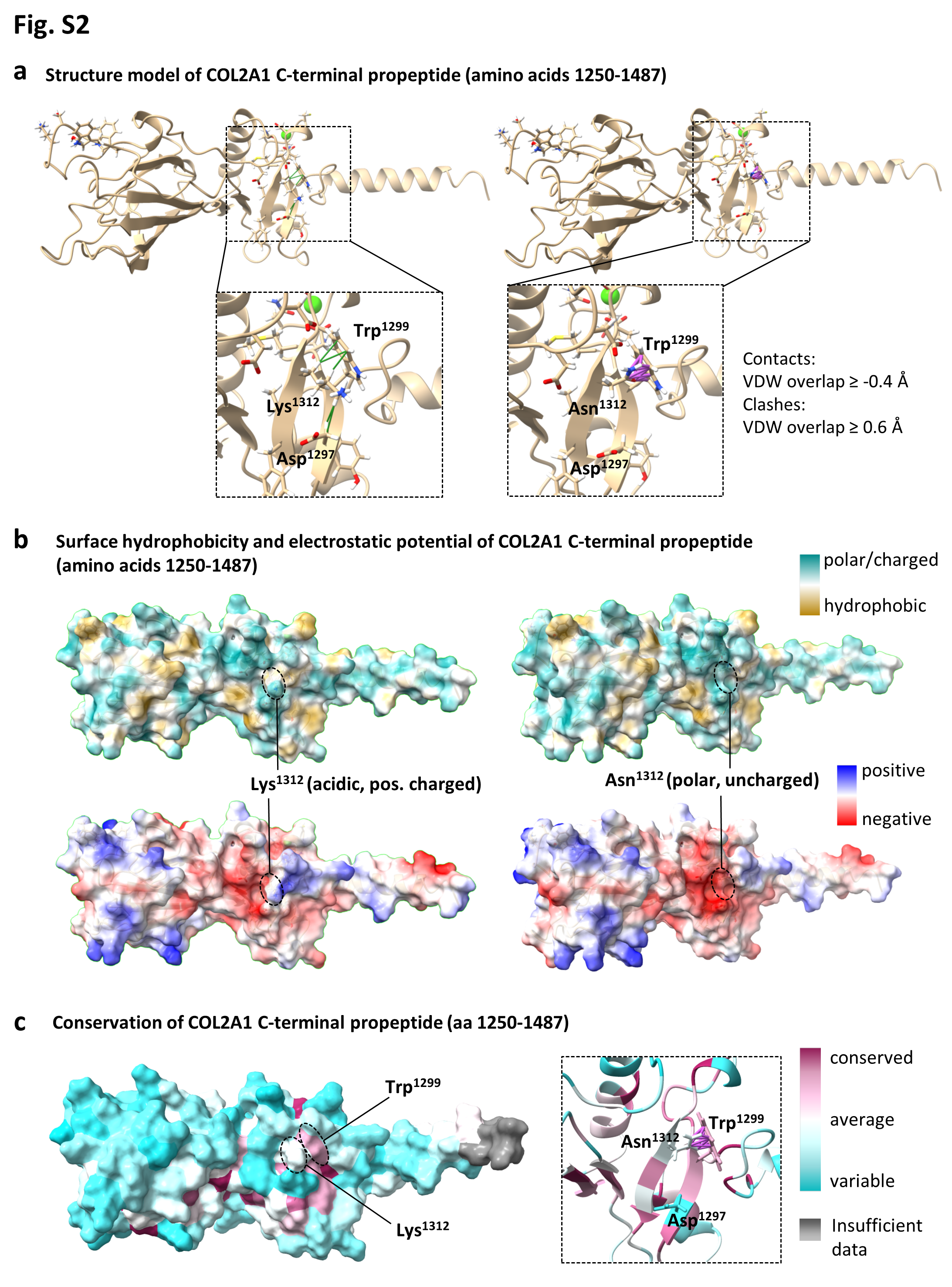

Supplement: Supplementary file 3 — Supplementary Figure S2. [file 41598_2022_8476_MOESM3_ESM.tif]

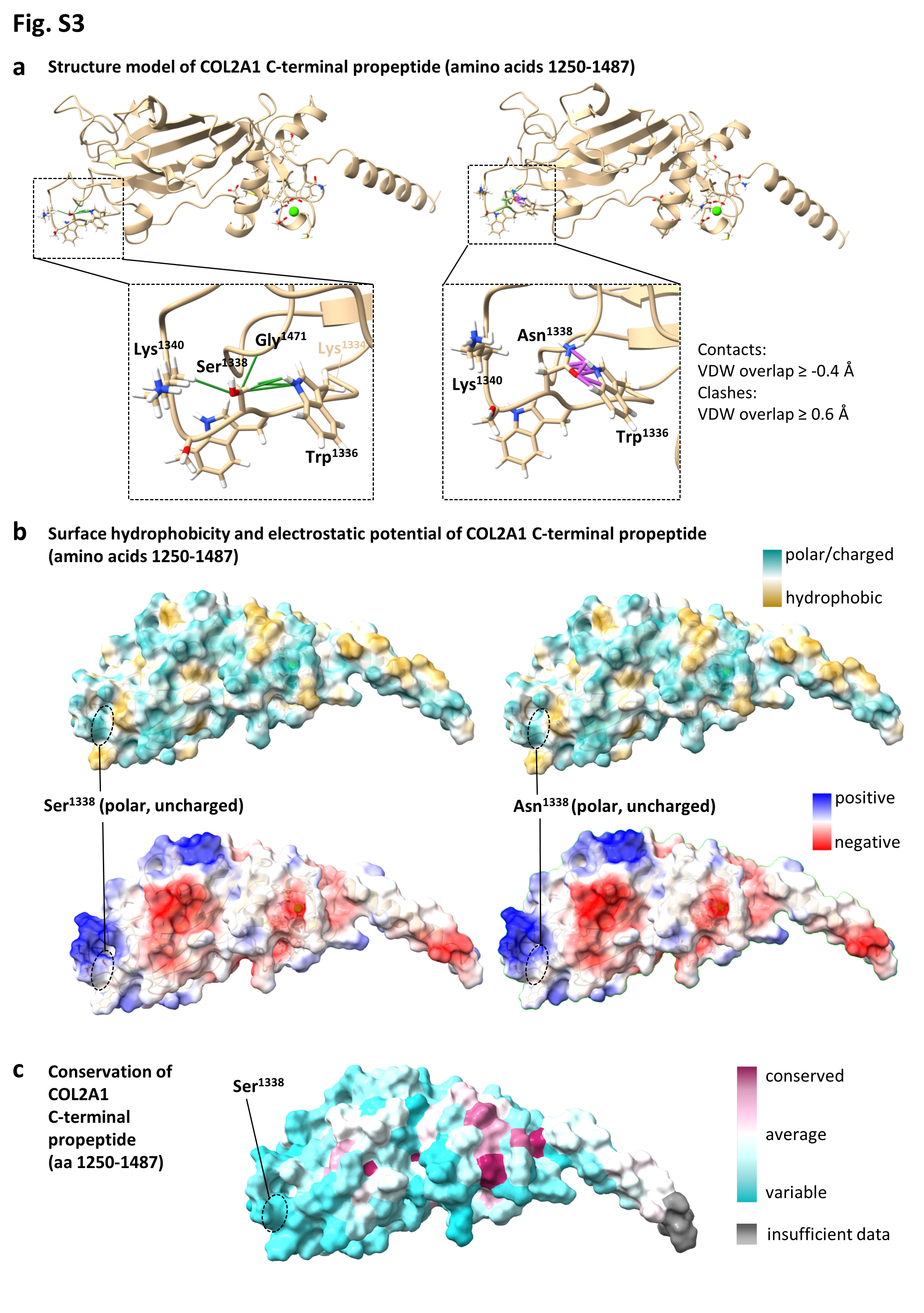

Supplement: Supplementary file 4 — Supplementary Figure S3. [file 41598_2022_8476_MOESM4_ESM.tif]

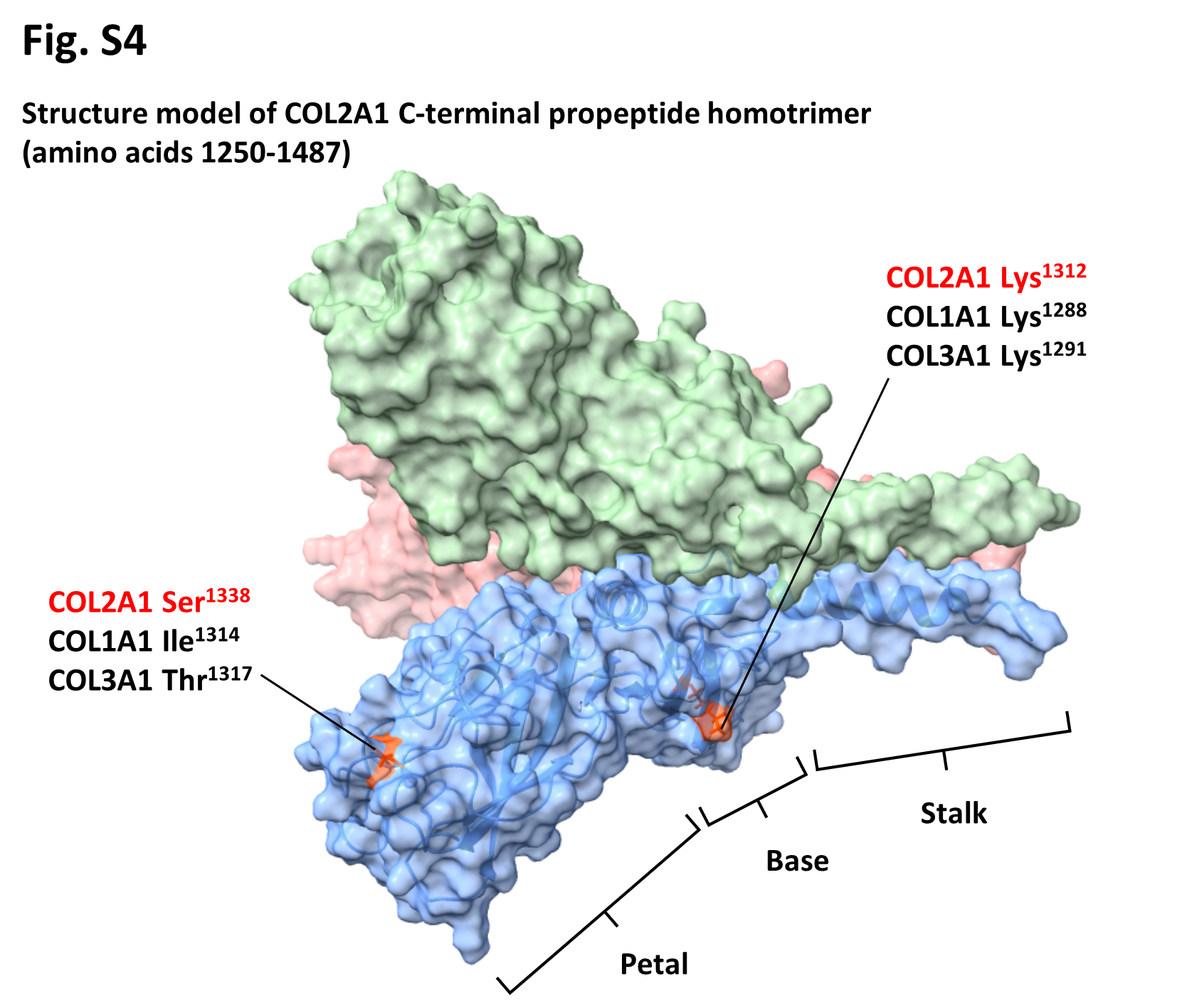

Supplement: Supplementary file 5 — Supplementary Figure S4. [file 41598_2022_8476_MOESM5_ESM.tif]
